# Comparison between germline and somatic loss-of-function RNF43 mutations reveals different genotype-phenotype associations and provides insights into the genetic mechanisms of colorectal tumourigenesis

**DOI:** 10.1136/gutjnl-2025-337030

**Published:** 2025-12-24

**Authors:** Claire Palles, Luke Freeman-Mills, Edward Arbe-Barnes, Nathalie Feeley, Laura Chegwidden, Helen Curley, Sara Galavotti, Connor Woolley, Jeremy Cheadle, Dmitri Mouradov, Oliver Sieber, Silvia Salatino, Steve Thorn, Anshita Goel, Juan Fernandez-Tajes, Sulochana Omwenga, Sujata Biswas, Timothy Maughan, Simon J Leedham, S:CORT Consortium, Andrew Blake, UK Colorectal Cancer Genomics Consortium, Alex J Cornish, CORGI Consortium, Kai Ren Ong, WGS500 Consortium, Peter Donnelly, Viktor Hendrik Koelzer, Lai Mun Wang, Roland Arnold, James Edward East, Ian Tomlinson

**Affiliations:** 1 Institute of Cancer and Genomic Sciences, University of Birmingham, Birmingham, UK; 2 Birmingham Biomedical Research Centre, UK, National Institute for Health and Care Research, Birmingham, UK; 3 Wellcome Centre for Human Genetics, University of Oxford, Oxford, UK; 4 Scotland Cancer Centre, University of Edinburgh, Edinburgh, UK; 5 Department of Oncology, University of Oxford, Oxford, UK; 6 School of Medicine, Cardiff University School of Medicine, Cardiff, UK; 7 Personalised Oncology Division, Walter and Eliza Hall Institute of Medical Research, Melbourne, Victoria, Australia; 8 Department of Medical Biology, The University of Melbourne, Melbourne, Victoria, Australia; 9 Personalised Oncology Division, Walter and Eliza Hall Institute of Medical Research, Parkville, Victoria, Australia; 10 Nuffield Department of Medicine, University of Oxford, Oxford, UK; 11 Translational Gastroenterology Unit, Univerity of Oxford Nuffield Department of Medicine, Oxford, UK; 12 Institute of Medical Genetics and Pathology, University Hospital Basel, Basel, Switzerland; 13 Computational and Translational Pathology Group, Department of Biomedical Engineering, University of Basel, Basel, Switzerland; 14 Department of Laboratory Medicine, Changi General Hospital, Singapore; 15 NIHR Oxford Health Biomedical Research Centre, Oxford, UK

**Keywords:** COLONIC POLYPS, COLORECTAL ADENOMAS, COLORECTAL CARCINOMA, GENETICS, INHERITED CANCERS

## Abstract

**Background:**

Germline *RNF43* mutations cause a dominantly inherited syndrome of colorectal cancer (CRC) and serrated polyps. However, these data originate from highly selected families.

**Objective:**

We assessed germline *RNF43* variants in patients more representative of the general population and compared these with somatic *RNF43*mutations in CRCs.

**Design:**

We studied 49 823 CRC and/or polyp cases from the CORGI study, 100 000 Genomes (100kGP) and UK Biobank (UKB), alongside 165 250 controls. Somatic mutations were analysed in 2722 CRCs.

**Results:**

Consistent with the literature, a germline loss-of-function *RNF43* variant (p.Thr158ProfsTer6) was found in a multigenerational CORGI family with early-onset CRC and serrated and/or filiform polyps. However, while 23 CRC/polyp cases and 47 controls from 100kGP or UKB had germline *RNF43* mutations, cases often lacked multiple polyps or a notable family history. Sometimes, CRCs developed independently of the germline *RNF43* mutation. In case-control analyses, germline *RNF43* variants were associated with CRC risk (OR=2.696, p=0.010), but penetrance was much greater for germline mutations in the N-terminal half of the gene. Germline C-terminal mutations conferred no increased CRC risk. However, somatic C-terminal mutations were pathogenic, perhaps because their relatively weak effects are supplemented by accompanying mutations in Wnt genes, including *ZNRF3* and a new driver, *SFRP4*.

**Conclusion:**

*RNF43* is a CRC predisposition gene, but risks are moderate, the reported polyposis phenotype is often absent and molecular phenocopies can occur. N-terminal germline *RNF43* variants confer higher risk, although weak effects of C-terminal variants cannot be excluded. Genetic testing and patient management should incorporate these factors.

**WHAT IS ALREADY KNOWN ON THIS TOPIC**

- Germline *RNF43* mutations reportedly cause a dominantly inherited syndrome of multiple serrated polyps and colorectal cancer (CRC). This is arguably the only established genetic cause of serrated polyposis syndrome (SPS). However, these data come from a handful of families, and cancer risks and optimal management are unclear.

**WHAT THIS STUDY ADDS**

- Germline *RNF43* mutations can cause phenotypes like those in the literature. However, some gene carriers develop CRC without multiple polyps, others develop CRC with no contribution from their germline *RNF43* mutation, and yet others develop neither CRC nor polyps; hence overall, the CRC risks are only moderately (2.7-fold) increased. N-terminal germline mutations cause a six-fold increased CRC risk, but mutations in the C-terminal half of the *RNF43* gene appear weakly or non-penetrant. By contrast, in MSI+ sporadic CRCs, *somatic* mutations throughout most of *RNF43* are pathogenic and act as classical tumour suppressor alleles. Their effects may be relatively weak, relying on available Wnt ligands and/or concomitant driver mutations in other Wnt genes, such as *ZNRF3, AXIN2* and *SFRP4*.

**HOW THIS STUDY MIGHT AFFECT RESEARCH, PRACTICE OR POLICY**

- Germline *RNF43* mutations are probably more common than previously thought, but the phenotype is highly variable and often unremarkable, plausibly because the risk of CRC depends on a separate polyp-forming risk. Efficient identification of gene carriers and risk management are both challenging.

## Introduction

 Activation of the Wnt pathway is arguably central to the growth of all colorectal cancers (CRCs).^1^,^3^ In sporadic CRCs, increased Wnt usually occurs through somatic loss-of-function (LoF) *APC* mutations, although about 10% of CRCs activate Wnt through gain-of-function mutations in beta-catenin *CTNNB1* or by LoF changes in *RNF43.*^4^ *RNF43* is a somatic driver gene in at least 11 cancer types (https://www.intogen.org/search?gene=RNF43).^5^ It encodes a 784-amino acid RING-type E3 ubiquitin ligase that degrades Frizzled Wnt receptors, leading to Wnt activation as long as sufficiently high levels of extra-cellular Wnt ligands are present.^6^,^8^ *RNF43* has several functional domains, including (N-terminus to C-terminus): transmembrane; protease-associated; ectodomain; cytoplasmic RING and C-terminal. While most, and perhaps all, pathogenic *RNF43* mutations cause at least partial loss of function, much remains unclear about their pathogenicity, including: the effects of mutations in different regions of the protein;^9^ the role of missense and splice variants;^10^ the sensitivity of different mutant RNF43 proteins to blockade of Wnt ligand production; and the importance of recurrent somatic frameshift mutations at hotspot sites such as codons 659 and 117,^9^ in cancers that are microsatellite-unstable (MSI+) owing to mismatch repair-deficiency.

**Figure 1:**
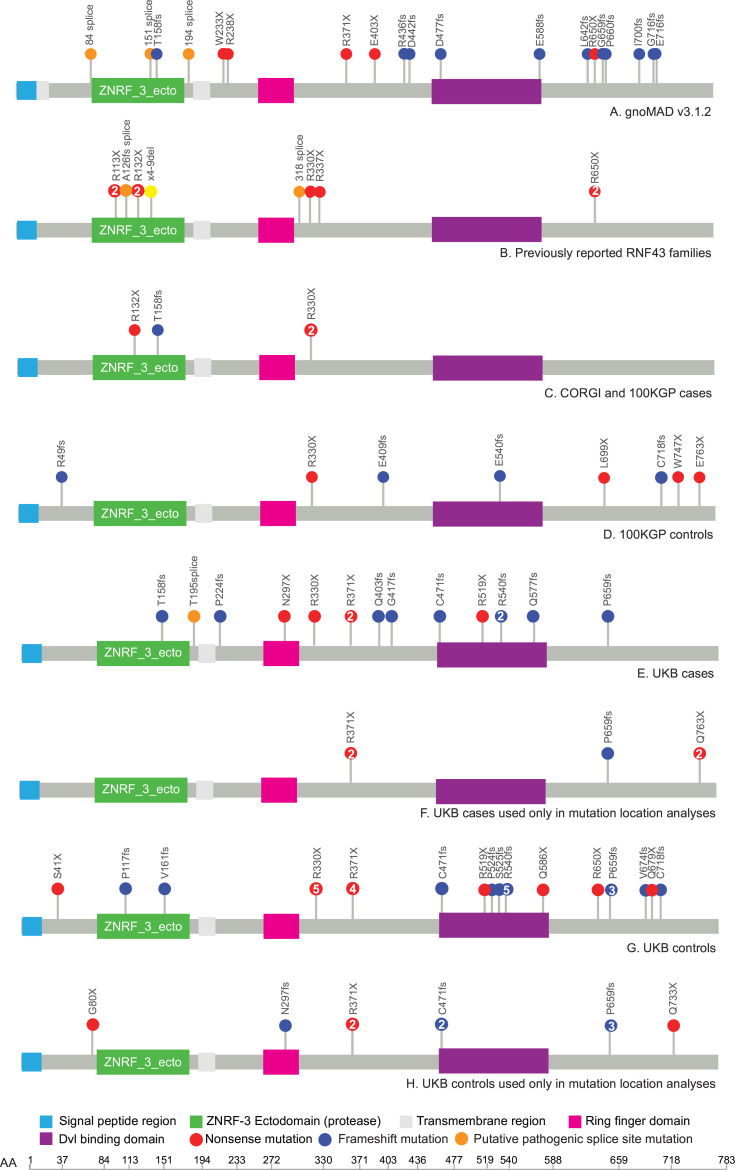
Lollipop plots showing amino acid positions (ENST00000407977.7) of all germline LoF *RNF43* variants from this study, previous reports in the literature and gnomAD. The following types of mutations were considered ‘LoF’: frameshift (fs), nonsense (X), large deletion, and splice donor and acceptor predicted to be pathogenic by SpliceAI (https://spliceailookup.broadinstitute.org/). Numbers of individuals with that genotype (if two or more) are displayed. No individual is shown twice. Domain boundaries are from.^43^ Plots were generated with SRPlot (http://www.bioinformatics.com.cn/plot_basic_lollipop_mutation_diagram_090_en). (A) gnomAD V.3.1.2 LoF variants in 76 156 individuals. Only the splice site change c.252+2C>G (near codon 84) and frameshift at codon 700 were from cancer cases. (B) Previously reported cases (online supplemental table 1). Probands were included as cases in mutation location analyses (table 3), except for one R337X carrier who was a control and one R650X carrier who was excluded based on no colorectal phenotype but a cancer of another site (online supplemental table 1). (C) CORGI and 100kGP cases with CRC and/or multiple polyps. Note that the two molecular phenocopy cases with germline R330X mutations are included with the cases here (and in table 1), even though their tumours developed independently of *RNF43*. (D) 100kGP controls. Note that the Q763X carrier shown here was only eligible for the mutation location analyses and was excluded from the case-control analysis based on ancestry. (E) UK Biobank (UKB) cases with International Classification of Disease (ICD)-10 coded colorectal polyps, colorectal adenomas or colorectal cancer. (F) UKB cases used only in mutation location analyses and excluded from the case-control analysis based on ancestry or relatedness. (G) UKB controls without any ICD-10 codes for colorectal cancer, colorectal adenomas or colorectal polyps and without personal or family history of cancer. (H) UKB controls used only in mutation location analyses and excluded from the case-control analysis based on ancestry or relatedness. 100kGP, 100 000 Genomes; CRC, colorectal cancer; LoF, loss-of-function.

In some of the Mendelian (high-penetrance) CRC predisposition syndromes,^11 12^ Wnt activation occurs directly via germline mutation in *APC,* or indirectly through hypermutation caused by defects in DNA repair. LoF *RNF43* mutations have previously been reported in 11 families, mostly recruited on the basis of unexplained, multiple colorectal polyps^1013^,^18^ (details in figure 1, online supplemental table 1 and item 1). There also remain clinically important numbers of individuals with colorectal tumours, whose phenotypes resemble Mendelian syndromes, but who do not have identifiable germline mutations in the known predisposition genes.

In this study, we initially searched for previously undetected, high-penetrance CRC predisposition genes in a set of CRC and/or multiple polyp cases that included a very large kindred with apparently dominant inheritance of CRC and/or polyps of an unusual morphology. After identifying a germline *RNF43* mutation in this family, we extended our search for germline *RNF43* mutations to larger sets of CRC and polyp patients and to control individuals. In a complementary analysis, owing to the rarity of germline *RNF43* variants, we profiled somatic *RNF43* mutations in patients with sporadic CRCs to investigate mutation-specific pathogenicity. Our overall findings support *RNF43* as a CRC predisposition gene, which we argue should be tested in the clinical setting, and as a classical somatic tumour suppressor gene (TSG) in sporadic cancers. However, we also identify genetic complexity that mandates cautious interpretation and careful management of individuals with germline *RNF43* variants.

## Results

**Figure 2:**
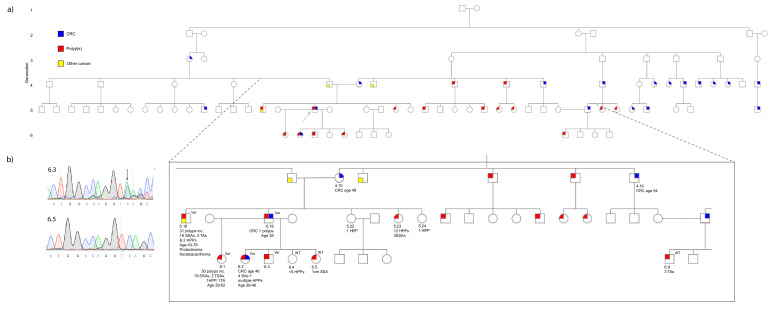
Pedigree of family Ox7. (A) Full pedigree and (B) branch of the family primarily analysed in this study. Medical notes and histopathology reports were obtained for as many family members as possible, with available details shown. CRCs, polyps and other cancers (which included prostate, bladder, ovarian and basal cell skin cancers) are shown in colour. Note that individuals with polyp phenotypes regarded as unremarkable, and hence possible phenocopies, are shown uncoloured (details in B). Patient mutation status (wildtype=+, mutation=Var) is shown. Sanger sequencing was used to validate whole-genome sequencing and genotype additional family members, and electropherograms showing variant and wildtype sequence are shown in B. Pedigree was drawn using information collected by the Oxford Genomic Medicine Service and CORGI study team with help from multiple family members. CRC, colorectal cancer; HPP, hyperplastic polyp; SA, serrated adenoma; SSA, serrated sessile adenoma; TA, tubiular adenoma.

**Figure 3:**
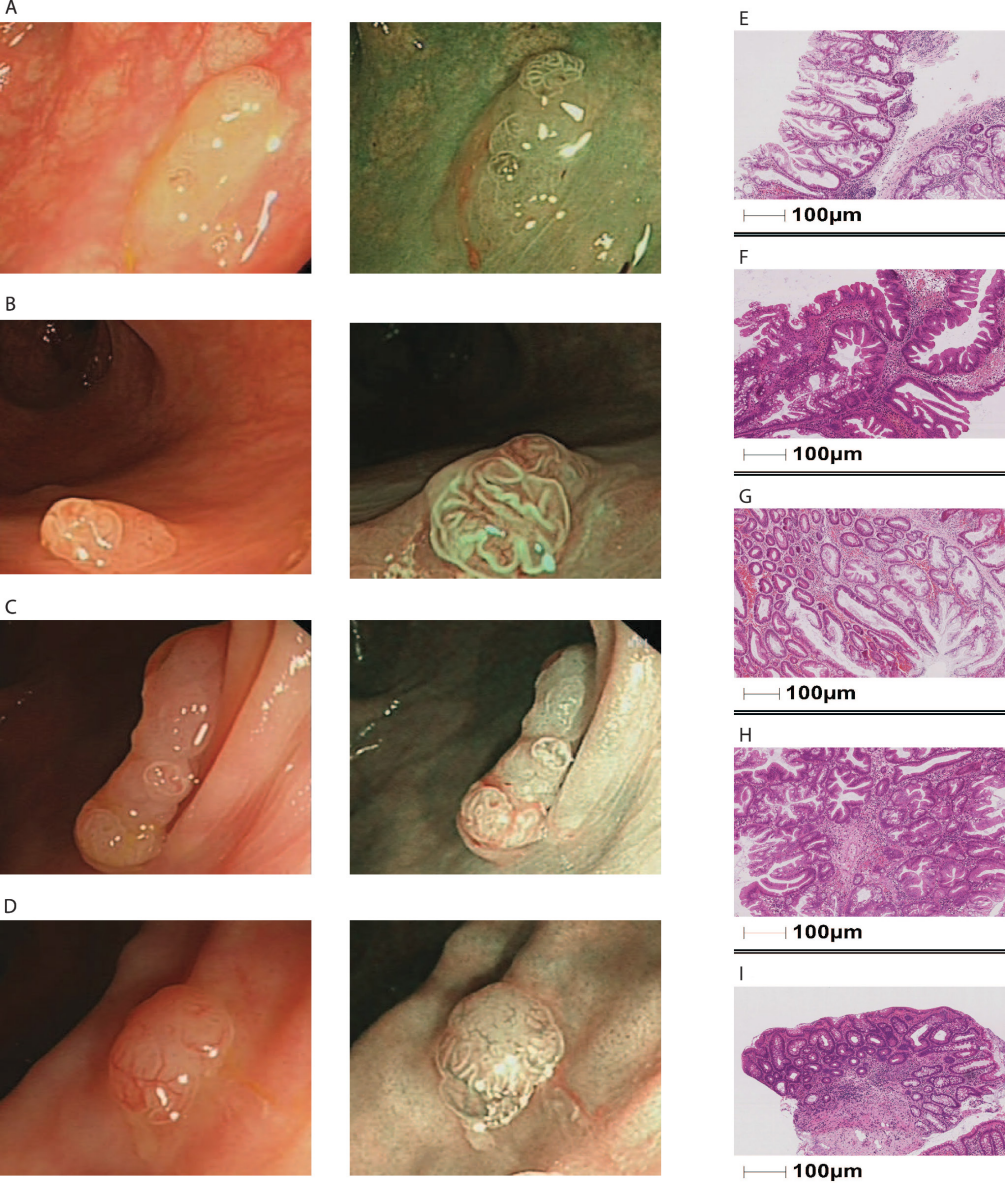
Colonoscopy images (**A–D**) and histology (**E–I**) of polyps from Ox7 (A–D) are paired white light images and narrow band images (using a blue light technique that highlights microvessels). Polyps ranged in size from 3 mm to 9 mm and, as can be seen, hyperplastic, adenomatous and serrated adenomatous features could be present within a single polyp. (E) sessile serrated lesion (SSL) with elongated crypts and serrated epithelium; (F) SSL with serrated low grade dysplasia and cells with prominent nuclei showing pseudostratification and hypereosinophilic cytoplasm; (G) low grade SSL with intestinal dysplasia resembling that of conventional adenomas with tubular architecture; (H) traditional serrated adenoma, with characteristic ectopic crypts and generalised cytologic dysplasia; (I) tubular adenoma with hyperchromatic basal nuclei, showing surrounding disorganised crypts with low goblet cell density. Images at additional magnifications are shown in online supplemental figure 1.

The CORGI study aims to identify CRC predisposition genes and variants based on familial colorectal cancer and/or multiple polyp cases from UK Clinical Genetics Centres. Forty cases initially underwent whole-genome sequencing (WGS) of constitutional DNA, including members of an exceptionally large, six-generation pedigree (Ox7) with apparently dominant inheritance of colorectal tumours (figure 2, online supplemental table 2). Affected individuals developed CRC and/or multiple colorectal polyps, some of which were reported as having filiform and/or serrated morphology (figure 3, online supplemental figure 1, online supplemental tables 2 and 3). Extracolonic tumours were usually absent or otherwise unremarkable, comprising single cases with carcinoma of the prostate, bladder, ovary or skin (basal cell carcinoma). The pedigree contained two loops owing to marriages between second cousins. The branch of the family shown in figure 2B was the focus of our study.

We performed WGS of constitutional DNA (online supplemental tables 1 and 2) and found that several family members carried germline variant *RNF43* ENSP00000385328.2:p.Thr158ProfsTer6; figure 1C). This variant, which was confirmed in the Regional Clinical Genetics Laboratory,^19^ was present once in gnomAD V.3.1.2 samples (total allele count=64 792, allele frequency=1.54×10^–5^; figure 1A), but was otherwise absent from the literature (figure 1B) and public databases. The variant was present in Ox7 individuals 5.16, 6.1, 6.2, 6.3 and by inference, 5.19 (figure 2). All these gene carriers had developed >10 polyps, and 5.19 and 6.2 had developed CRC. On review by VHK and LMW, polyp morphology was predominantly reported as sessile serrated lesions (SSLs), hyperplastic polyps (HPPs) and tubular adenomas (TAs) (online supplemental table 3). Some non-gene carriers 6.4, 6.5 and 6.9, who had been undergoing yearly screening colonoscopy owing to their family history, had also developed polyps, respectively, from their records <five HPPs, one 1 cm diameter SSL and three TAs (online supplemental table 2).

### Somatic changes in tumours from carriers of germline RNF43 p.Thr158ProfsTer6

Up to nine polyps from one member of Ox7 were available for molecular analyses (online supplemental table 4). A second hit at *RNF43* by copy-neutral loss of heterozygosity (LOH) or somatic mutation was found in five of seven (71%) polyps analysed (online supplemental figure 2), and there was no evidence of second hits by methylation (online supplemental table 5). No pathogenic somatic mutations were found in the other major Wnt drivers *APC* and *CTNNB1* (online supplemental table 6), but most polyps (five of seven) had somatic driver mutations in *BRAF* or *KRAS* (online supplemental figure 2). All seven polyps analysed were mismatch repair-proficient/microsatellite-stable (MSI-negative, online supplemental table 7), and all four polyps analysed for DNA methylation were CpG Island Methylator Phenotype (CIMP)-negative (online supplemental table 8).

Four polyps were analysed by RNA sequencing, and differential expression analysis was performed against 53 sporadic polyps from the S:CORT project. Online supplemental figure 3 shows that, based on expression of Wnt pathway genes (online supplemental table 9), the Ox7 polyps clustered together close to SSLs and traditional serrated adenomas (TSAs). Furthermore, as expected, given that mutant *RNF43* requires the presence of Wnt ligands to have an effect, we did not detect over-expression of negative feedback regulators of Wnt (online supplemental figure 4), consistent with ligand-dependent Wnt pathway activation in the Ox7 polyps.^20^

### Germline LoF RNF43 variants in the CORGI study, 100 000 Genomes Project and UK Biobank

**Figure 4:**
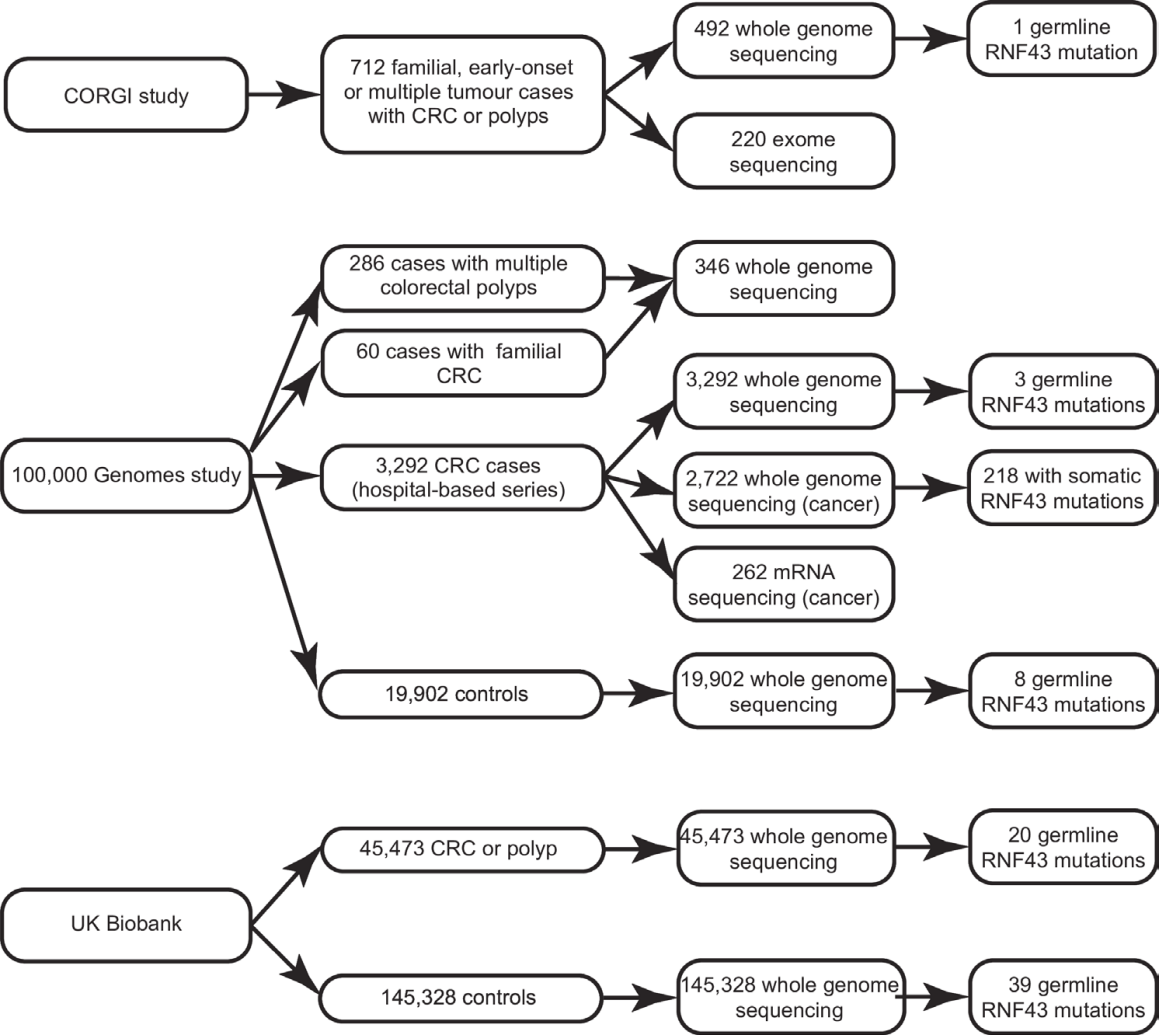
CORGI, 100 000 Genomes and UK Biobank patients and tumours. The numbers of individuals are provided prior to some of the filtering steps used for the analysis of CRC/polyp risk in the case-control study or the effects of mutation location within the *RNF43* gene on risk (eg, exclusions based on non-European ancestry, relatedness, age and presence of other cancers or cancer-associated conditions). The final numbers and individuals included in the association and mutation location studies are shown in tables 1–3 and figure 1. CRC, colorectal cancer.

We extended the search for germline *RNF43* mutations to nearly 50 000 UK cases with CRC and/or polyp(s) who had undergone WGS (see the Methods section, online supplemental tables 10 and 11, figure 4). We identified and excluded 21 individuals with extracolonic cancers (online supplemental table 12). No further CORGI or 100 000 Genomes (100kGP) Rare Disease (Cancer) Domain cases harboured pathogenic *RNF43* variants, but three patients from the 100kGP CRC Domain had protein-truncating germline mutations in *RNF43* (table 1, figure 1C). One of these individuals carried *RNF43* c.394C>T; p.Arg132Ter, presenting with a T3N1 carcinoma of the sigmoid colon in her eighth decade, which was sequenced. A single polyp was also found in her ninth decade. However, there was no recorded family history of colorectal or other tumours. The cancer was MSI-negative, had a second hit at *RNF43* by copy-neutral LOH and, consistent with *RNF43* pathogenicity, harboured no somatic mutations in the major Wnt driver genes that are functional alternatives to *RNF43,* including *APC, CTNNB1* and R-spondin fusions. Two unrelated 100kGP CRC patients without reported polyps carried the LoF germline *RNF43* mutation, c.988C>T;p.Arg330Ter, which is annotated as pathogenic by Clinvar (https://www.ncbi.nlm.nih.gov/clinvar/variation/932427/?new_evidence=true) and has been assigned as the cause of serrated polyposis syndrome (SPS) in a relatively large family.^17^ Unexpectedly, however, neither of these patients’ tumours had second hits at *RNF43*. Instead, both tumours had acquired bi-allelic, pathogenic somatic *APC* mutations. The first patient’s cancer was MSI+ and occurred in the sixth decade, and the second was MSI-negative, presenting in the ninth decade. These two individuals could thus be termed ‘molecular phenocopies’, in distinction from simple phenocopies (who are generally sporadic cases, affected by cancer who do not carry a predisposition gene that is present within their family). One further case from UK Biobank (UKB) and six controls from 100kGP or UKB were also p.Arg330Ter carriers (table 1; figure 1). Our data therefore raised concerns that p.Arg330Ter germline mutations, and perhaps other germline LoF *RNF43* variants, were benign or had incomplete penetrance.

**Table 1:** *RNF43* carriers by study and case/control status for case-control association study

ID	Origin	Case/control	Age	Sex	Medical history colorectal	Family history (1^o^ relative)	Germline variant	Pathogenic?
1	CORGI	Case	39	M	CRC, SPS	CRC and polyposis	p.Thr158ProfsTer6	VUS
2	100kGP	Case	75–85	F	CRC	None	p.Arg132Ter	P
3	100kGP	Case	55–65	F	CRC (phenocopy)	None	p.Arg330Ter	LP
4	100kGP	Case	75–85	F	CRC (phenocopy)	None	p.Arg330Ter	LP
1	100kGP	Control	50–60	M		None	p.Arg49SerfsTer25delAG	NA
2	100kGP	Control	35–45	M		None	p.Arg330Ter	LP
3	100kGP	Control	50–55	M		None	p.Gln409ProfsTer36	LP
4	100kGP	Control	60–70	F		None	p.Gly540ArgfsTer56	NA
5	100kGP	Control	10–20	F		None	p.Leu699Ter	NA
6	100kGP	Control	40–50	M		None	p.Cys718LeufsTer28	NA
7	100kGP	Control	35–45	F		None	p.Trp747Ter	NA
1	UKB	Case	60s	F	Polyps	None	p.Thr158ProfsTer6	NA
2	UKB	Case	70s	F	CRC, polyp(s), anal cancer	CRC mother and father	c.583–1G>T	CP
3	UKB	Case	60s	M	Polyps	Ca. prostate, father	p.Pro224AlafsTer200	CP
4	UKB	Case	70s	M	Polyp(s)	None	p.Asn297Ter	CP
5	UKB	Case	60s	F	CRC	CRC mother	p.Arg330Ter	LP
6	UKB	Case	50s	F	CRC, polyps	None	Arg371Ter	CP
7	UKB	Case	50s	M	Polyps	CRC mother, sibling	Arg371Ter	CP
8	UKB	Case	60s	M	CRC, polyps, ca. prostate	None	p.Gln403Ter	CP
9	UKB	Case	50s	M	Polyps	None	p.Gly417AspfsTer2	NA
10	UKB	Case	60s	M	Polyps, cholangiocarcinoma	Lung cancer sibling	p.Cys471ValfsTer31	VUS
11	UKB	Case	50s	F	Polyps	None	p.Arg519Ter	VUS
12	UKB	Case	50s	M	Polyp(s)	None	p.Gly540ArgfsTer56	NA
13	UKB	Case	50s	M	Polyp	None	p.Gly540ArgfsTer56	NA
14	UKB	Case	60s	M	Polyps	None	p.Gln577SerfsTer123	NA
15	UKB	Case	60s	M	CRC	None	p.Pro659SerfsTer87	LP
1	UKB	Control	70s	F		None	p.Ser41Ter	LP
2	UKB	Control	50s	M		None	p.Arg117ProfsTer8	CP
3	UKB	Control	60s	M		None	p.Val161SerfsTer7	NA
4	UKB	Control	60s	F		None	p.Arg330Ter	LP
5	UKB	Control	60s	M		None	p.Arg330Ter	LP
6	UKB	Control	60s	F		None	p.Arg330Ter	LP
7	UKB	Control	70s	M		None	p.Arg330Ter	LP
8	UKB	Control	60s	F		None	p.Arg330Ter	LP
9	UKB	Control	70s	M		None	Arg371Ter	CP
10	UKB	Control	70s	M		None	Arg371Ter	CP
11	UKB	Control	50s	F		None	Arg371Ter	CP
12	UKB	Control	70s	M		None	Arg371Ter	CP
13	UKB	Control	70s	F		None	p.Cys471ValfsTer31	VUS
14	UKB	Control	60s	M		None	p.Arg519Ter	VUS
15	UKB	Control	70s	M		None	p.Pro524LeufsTer3	NA
16	UKB	Control	60s	M		None	p.Ser525ValfsTer2	NA
17	UKB	Control	70s	M		None	p.Gly540ArgfsTer56	NA
18	UKB	Control	70s	F		None	p.Gly540ArgfsTer56	NA
19	UKB	Control	60s	M		None	p.Gly540ArgfsTer56	NA
20	UKB	Control	50s	M		None	p.Gly540ArgfsTer56	NA
21	UKB	Control	60s	M		None	p.Gly540ArgfsTer56	NA
22	UKB	Control	60s	M		None	p.Gln586Ter	NA
23	UKB	Control	60s	F		None	p.Arg650Ter	CP
24	UKB	Control	50s	F		None	p.Pro659SerfsTer87	LP
25	UKB	Control	80s	F		None	p.Pro659SerfsTer87	LP
26	UKB	Control	80s	M		None	p.Pro659SerfsTer87	LP
27	UKB	Control	50s	F		None	p.Val674SerfsTer70	VUS
28	UKB	Control	60s	M		None	p.Gln679Ter	NA
29	UKB	Control	70s	F		None	p.Cys718LeufsTer28	VUS

Inclusion criteria for case-control analyses are detailed in the Methods section. For family Ox7, a single entry is provided in this table (details in online supplemental table 2Supplementary Table 2). Details of tumour histology, where available, are shown in online supplemental table 3Supplementary Table 3, but with the exception of Ox7, a florid polyposis was absent from our cases’ records. Age at presentation is shown by decade in 100kGP to preserve confidentiality according to ethical permissions. Note that we did not formally assess CRC or polyp penetrance by age, given the different recruitment criteria and methods of each study. Family history of colorectal and other tumours, and other relevant major conditions is shown. ‘Pathogenic?’: pathogenicity from Clinvar V.12.5.25. Empty cells indicate inapplicable or unavailable. These individuals’ mutations, together with those of other selected groups (eg, previously published studies, gnomAD database), are shown in figure 1.

CP, conflicting pathogenicity; CRC, colorectal cancer; 100kGP, 100 000 Genomes; LP, likely pathogenic; NA, not assessed; P, pathogenic; SPS, serrated polyposis syndrome; UKB, UK Biobank; VUS, uncertain significance.

In total, 23 CRC/polyp cases and 47 controls from 100kGP or UKB carried LoF germline *RNF43* variants (figure 1). This low yield of carriers from those with polyps or CRC is consistent with other recent findings.^21^ One of the CRC/polyp cases from UKB carried the same variant observed in Ox7 and a further carrier was identified in UKB who was excluded from the control series because of a diagnosis of bladder cancer. To determine whether germline *RNF43* variants increased colorectal tumour risk, we performed association analyses (table 2), having excluded related individuals and those of non-European ancestry (see the Methods section). We found no significant association with the risk of CRC and/or polyp(s) (OR 1.615, 95% CI 0.927 to 2.815, p=0.091), but a stronger, significant association with CRC risk alone (OR=2.696, 95% CI 1.269 to 5.727, p*=*0.010).

**Table 2:** Association between germline *RNF43* mutations and (1) CRC and/or colorectal polyps or (2) CRC (with or without polyps)

(a) CRC and/or polyp(s)					
Study	N RNF43-mutant cases/total	N *RNF43*-mutant controls/total	OR	95% CI	P value
100kGP+CORGI	4/3484	7/14 432	2.369	0.508 to 9.322	0.242
UK Biobank	15/44 995	29/128 624	1.479	0.737 to 2.851	0.229
Meta-analysis			1.615	0.927 to 2.815	0.091
*Phet=*0.50; I^2^ 70%					

(b) CRC					
Study	N *RNF43*-mutant cases/total	N *RNF43*-mutant controls/total	OR	95% CI	P value
100kGP+CORGI	4/3036	7/14 432	2.719	0.583 to 10.701	0.108
UK Biobank	5/8275	29/128 624	2.681	0.810 to 7.007	0.052
Meta-analysis			2.696	1.269 to 5.727	0.010
*Phet=*0.99; I^2^ 0%					

(c) CRC and/or polyp(s), only mutations before or at codon 330					
Study	N *RNF43*-mutant cases/total	N *RNF43*-mutant controls/total	OR	95% CI	P value
100kGP+CORGI	4/3484	2/14 432	8.293	1.188 to 91.689	0.0153
UK Biobank	5/44 995	8/128 624	1.787	0.460 to 6.195	0.342
Meta-analysis			2.814	1.153 to 6.863	0.023
*Phet=*0.14; I^2^ 54.4%					

(d) CRC, only mutations before or at codon 330					
Study	N *RNF43*-mutant cases/total	N *RNF43*-mutant controls/total	OR	95% CI	P value
100kGP+CORGI	4/3036	2/14 432	9.518	1.363 to 105.238	0.010
UK Biobank	2/8275	8/128 624	3.887	0.402 to 19.523	0.119
Meta-analysis			6.240	2.079 to 18.731	0.001
*Phet=*0.44; I^2^ 0%					

(e) CRC, only mutations before codon 330					
Study	N *RNF43*-mutant cases/total	N *RNF43*-mutant controls/total	OR	95% CI	P value
100kGP+CORGI	2/3036	1/14 427	9.513	0.495 to 561.206	0.080
UK Biobank	1/8275	3/128 624	5.182	0.099 to 64.538	0.221
Meta-analysis			7.301	1.437 to 37.101	0.017
*Phet=*0.71; I^2^ 0%					

(f) CRC, only mutations after codon 330					
Study	N RNF43-mutant cases/total	N RNF43-mutant controls/total	OR	95% CI	P value
100kGP+CORGI	0/3036	5/14 432			0.080
UK Biobank	3/8275	21/128 624	2.221	0.424 to 7.441	0.221
Pooled analysis*	3/11 311	26/143 056	1.459	0.283 to 4.763	0.468
*Phet=*0.71; I^2^ 0%					

Individuals with European ancestry were included in the study and close relatives were excluded (see the Methods section). Tables (c–e) show analyses when individuals with mutations in the C-terminal part of the gene were excluded. For (e), the two ‘molecular phenocopy’ CRC cases and a control, all with p.Arg330Ter mutations, were excluded. (f) shows analysis based only on C-terminal mutations. P values for individual studies are from two-tailed Fisher’s exact tests. Meta-analyses used the Mantel-Haenszel method.

* Pooled analysis owing to zero count in one cell.

CRC, colorectal cancer; 100kGP, 100 000 Genomes.

Further exploration showed that cases were more likely than controls to carry germline *RNF43* mutations towards the N-terminal of the protein, prior to codon 330 which lies close to the end of the RING finger domain (table 3; figure 1). We therefore repeated the association analysis assuming that only germline mutations at or before codon 330 were pathogenic. We observed significant associations with the risk of CRC and/or polyps (table 2), with ORs higher than those detected for all LoF variants. C-terminal mutations (after codon 330) were not associated with increased CRC risk (OR 1.459, 95% CI 0.283 to 4.763, p*=*0.468). With the caveat that this assessment is not fully population-based, we conclude that *RNF43* is probably a moderate risk CRC predisposition gene, perhaps akin to *PMS2*,^22 23^ if germline mutations are relatively close to the N-terminus of the protein, whereas effects of mutations towards the C-terminal are very limited or absent.

**Table 3:** Germline *RNF43* mutations in the proximal part of the gene are more prevalent in CRC and polyp cases than controls

(a) CRC and/or polyp(s)				
Codon	1–329	330	331–	Total
Cases	13	4	16	33
Controls	6	6	33	45
Total	19	10	49	78
p=0.028, Fisher’s exact, 2×3 table				

(b) CRC				
Codon	1–329	330	331–	Total
Cases	7	4	4	15
Controls	6	6	33	45
Total	14	10	37	60
P=0.003, Fisher’s exact, 2×3 table				

(a) Data from CRC and/or polyp cases are derived from this study and previously published data from individual cases and families with germline mutations (table 1, online supplemental table 1). Penetrance in Mendelian dominant CRC syndromes frequently varies within families and is detected across ancestry groups, with very few specific causes established. We therefore did not exclude mutation carriers according to ancestry or relatedness, although we set a minimum age of 50 years for controls. For familial cases, one individual per family (the nominal proband) was included in the data set. Based on our initial finding of 100kGP CRC patients with germline LoF bystander mutations at codon 330, we divided mutation position into pre-codon 330, codon 330 and post-codon 330. Other studies may define the C-terminal domain slightly differently, as the protein distal to the Dvl binding domain. The tendency for controls to have mutations after codon 330 was significant. (b) As for (a) but excluding cases with polyps only.

CRC, colorectal cancer; LoF, loss-of-function.

### Analysis of CRC genomes indicates that somatic mutations in the C-terminal region of RNF43 are frequently pathogenic

Our germline analyses raised the possibility that not all protein-truncating *RNF43* variants are pathogenic, especially those that occur later in the gene that might preserve important functions. This echoed previous suggestions that the highly recurrent somatic *RNF43* mutations at codon 659 (p.Pro659SerfsTer87, 659fs), close to the C-terminus, are bystanders arising from replication errors at a short tandem repeat in mismatch repair-deficient cancers.^24^ While somatic and germline mutations in the same gene cannot simply be regarded as equivalent, somatic *RNF43* mutation data in CRC are much more substantial than germline data and may thus provide important lessons on variant pathogenicity.

Previous studies of somatic *RNF43* mutations are outlined in online supplemental item 2. Some of these, based on exome sequencing, may have been prone to under-calling of indels in simple repeats (https://www.cbioportal.org/).^25^ We therefore investigated sporadic CRCs that had undergone WGS in the 100kGP. Of 2722 CRCs in 100kGP V.18, 620 were MSI+ and 2016 MSI-negative, with the remainder of unknown MSI status. 314, 39 and nine tumours of each type, respectively, carried one or more somatic LoF *RNF43* mutations (online supplemental figure 5). We identified putative pathogenic *RNF43 genotypes*, comprising bi-allelic protein-truncating mutations or mono-allelic mutations plus LOH. Importantly, where possible, we assessed the former as homozygous or heterozygous, since a substantial proportion of MSI+ CRCs acquired two independent, identical mutations in repeat sequences, such as those at codon 117 and 659, thereby plausibly creating a pathogenic genotype. Pathogenic *RNF43* genotypes occurred in 8% of all CRCs, comprising 188 MSI+, 25 MSI-negative and five other tumours. In MSI+ CRCs, homozygous or compound heterozygous frameshift mutations were the most frequent genotypes, occurring in >90% of tumours with bi-allelic LoF mutations, whereas nonsense or frameshift mutations accompanied by LOH were most frequent in MSI-negative tumours (n=22/25, 88%). In 48 tumours, a single *RNF43* mutation could not be classified confidently as homozygous or heterozygous (see the Methods section) and the genotypes of those tumours were denoted as of uncertain zygosity and pathogenicity.

**Figure 5:**
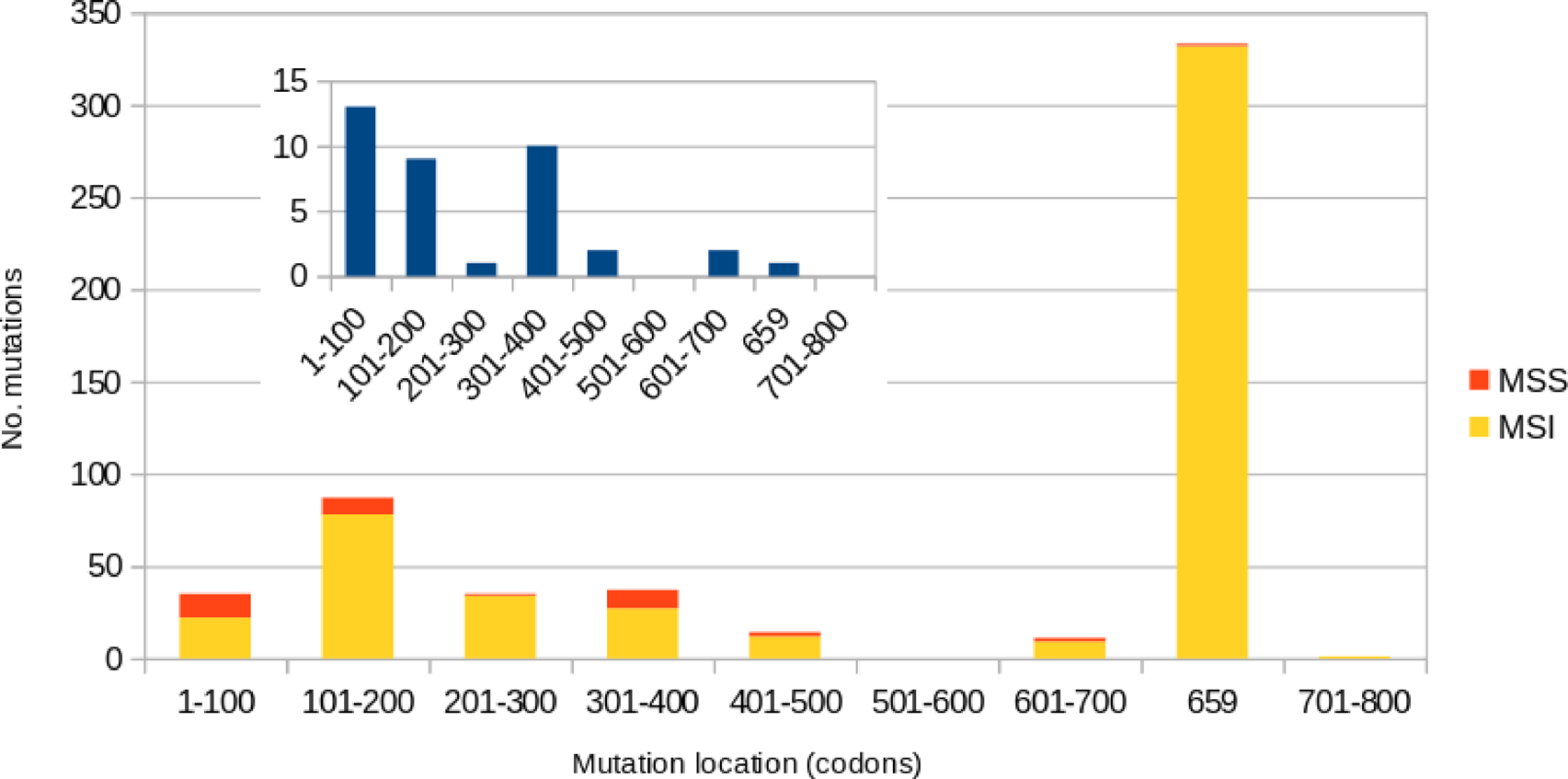
Locations of pathogenic somatic mutations in *RNF43* in 100kGP CRCs. The inset shows non-MSI tumour data in more detail. 100kGP, 100 000 Genomes; CRC, colorectal cancer.

659fs changes were by far the most common somatic mutation (333/553, 60%), followed by codon 117fs (48/553, 8.7%), both changes comprising small indels in short coding repeats. Aside from codon 659 changes, somatic mutations became less common after codon 350, distal to the RING domain of RNF43 (figures 1 and 5), and none was found between codons 500 and 600. The excess of putative bi-allelic over monoallelic mutations (118 v 59) did not support the previously postulated dominant negative model of *RNF43* mutation pathogenicity.^26 27^ Our data also did not support another specific mechanism for 659fs pathogenicity, namely nonsense-mediated mRNA decay, which is predicted to affect most LoF mutations before about codon 750, since the final *RNF43* exon encodes only amino acids 770–784. We assessed whether this was the case in 32 CRCs with somatic RNA-seq and WGS data. In agreement with previous studies,^28 29^ we found almost all LoF alleles, including 659fs, to produce a stable transcript. In some tumours, transcripts with LoF mutations were even slightly increased over the wildtype species, but there was large variation that prevented the drawing of any general conclusions regarding the relative stability of mRNA from wildtype and protein-truncating alleles (online supplemental figure 6). Overall, our data were consistent with previous evidence that C-terminal truncated *RNF43* mRNA and protein are stable.

We tested our observed *RNF43* somatic mutation data (only) against the following predictions that should hold if 659fs (and other mutations after codon 600) are *non-pathogenic*:

1. 659fs and nearby LoF mutations should be very rare in MSI-negative tumours.
2. The distribution of the three 659fs genotypes in MSI+ tumours should follow a binomial based on allele frequency.
3. The proportions of 659fs/+ and 659fs/659fs *RNF43* genotypes should be the same in MSI+ tumours with or without pathogenic *APC* or *CTNNB1* genotypes.
4. 659fs/659fs and 659fs/+ MSI+ tumours should be equally likely to have concomitant pathogenic *APC* or *CTNNB1* genotypes.
5. 659fs mutations should co-occur with other *RNF43* mutations at the same frequency, independent of whether the other mutations cause a pathogenic or non-pathogenic *RNF43* genotype.

In brief, while prediction (1) was validated, we refuted predictions (2)–(5). Details of these analyses are shown in online supplemental tables 13–15. The data thus supported the pathogenicity of somatic 659fs mutations.

A further prediction if 659fs changes are passengers is that 659fs/+ and 659fs/fs cancers should have another source of Wnt (if the generally accepted notion that all CRCs require Wnt activation is correct). We examined expression of *AXIN2*, which is known to be higher in CRCs carrying Wnt ligand-independent driver mutations, such as *APC* and *CTNNB1*, than in tumours with ligand-dependent drivers such as *RNF43* and R-spondin fusions.^20^ The *AXIN2* expression levels of tumours with bi-allelic 659fs mutations or a single 659fs and another LoF change were lower than bi-allelic *APC* mutant tumours (with concomitant *RNF43* mutation(s)) and than the large set of *RNF43-*wildtype tumours (mostly *APC* mutant). Thus, these data suggest that either the 659fs mutation is pathogenic, or the tumours concerned are both prone to acquire 659fs and also have an alternative, unidentified source of Wnt ligands (online supplemental figure 7).

*RNF43*’s functional homologue, *ZNRF3,* is also a CRC driver and is mutated in ~5% of tumours. *ZNRF3* mutations tended to co-occur with *RNF43* mutations overall (online supplemental figure 8), but there was no evidence that *ZNFR3* mutations could be the real cause of Wnt activation in 659fs mutants. Indeed, there was no difference in *ZNRF3* mutation frequency in 659fs-mutant compared with other *RNF43*-mutant CRCs (p*=*0.560). We therefore performed a hypothesis-free search for unidentified Wnt driver mutations in the set of 162 CRCs for which 659fs changes (or other nearby changes) were the only *RNF43*-inactivating mutation. Interestingly, this search detected not only several known CRC drivers, but also a novel candidate driver, the Wnt pathway gene *SFRP4,* which harboured a large variety of missense mutations in 33/162 (21%) of the 659fs-mutant cancers (online supplemental table 16). Whether these *SFRP4* changes could lead on their own to Wnt activation and hence render 659fs redundant was highly uncertain, since *SFRP4* was mutated just as frequently in cancers with non-659fs *RNF43* mutations (p*=*0.64). Any functional effect might thus reflect modulation of Wnt activity (eg, a shifted balance between canonical and non-canonical signalling), specifically in *RNF43*-mutant cancers.^30^ No other novel candidate Wnt drivers were found, although we could not exclude ligand over-expression or Wnt TSG silencing by methylation.

Overall, the literature review and our own analysis of somatic *RNF43* mutations indicated that *RNF43* is a TSG, generally inactivated in CRCs by LoF mutations and sometimes by LOH. While there is a decreased prevalence of somatic mutations in the C-terminal region of the gene, most evidence supported the pathogenicity of 659fs mutations and, by extension, of similar mutations nearby. However, because the short tandem repeat at codon 659 is highly prone to indels, we find that 659fs can sometimes be a passenger, acting as a superfluous ‘third hit’ to two more proximal *RNF43* mutations or to bi-allelic *APC* mutations (online supplemental figure 5).

## Discussion

There is a limited amount of pre-existing evidence that germline *RNF43* mutations predispose to a syndrome of CRC and multiple polyps. This is mostly derived from studies that are highly enriched for cases likely to have a genetic origin. Nevertheless, the limited data and associated uncertainty about the pathogenicity of many *RNF43* variants have meant that some centres still do not routinely include *RNF43* in clinical diagnostic panels. While our large patient set includes individuals selected for ‘genetic’ features, most participants in this study can be considered to more closely resemble sporadic cases of CRC and/or polyp(s) (although some degree of selection for CRC cases sequenced as part of 100kGP cannot be ruled out, given recruitment was largely from tertiary referral centres and academic centres). Performing association studies of rare germline alleles can be challenging owing to factors such as phenocopies, non-penetrance, suboptimal power and wide CIs for risk estimates. Nevertheless, we find that germline *RNF43* mutations moderately raise CRC risk in our patient sets, with a higher risk and penetrance for N-terminal mutations. C-terminal mutations may have limited or no effects on risk. Associations with polyp risk are less clear, perhaps reflecting the large ‘background’ burden of both classical adenomas and HPPs in the general population.

In our data from the single large Ox7 kindred, the phenotype associated with germline *RNF43* mutations seemed to be restricted to colorectal tumours. Colorectal cancer was also the most commonly observed cancer in carriers identified from the 100kGP and UKB. However, since *RNF43* is a somatic driver gene in malignancies of the pancreas, colorectum, endometrium, oesophagus, prostate, stomach and gall bladder,^531^,^35^ hypothetical risks of extra-colonic cancers should be borne in mind. Cancer types observed in *RNF43* LoF mutation carriers who did not meet the criteria for controls in our analyses of UKB and 100kGP data are shown in online supplemental table 12. Where present, the colorectal polyp phenotype in our gene carriers was mixed, being variously generally described as serrated lesions, sometimes with adenomatous features, classical adenomas and HPPs. However, the filiform lesions observed in our large family Ox7 raised the possibility that a more unusual, and perhaps specific, polyp morphology can also occur. Most strikingly, while we acknowledge that polyps may be under-reported in both 100kGP and UKB, both of those studies contain data fields for colorectal polyps and colonoscopies and sigmoidoscopies. The familial, multiple polyp phenotype previously associated with germline *RNF43* mutations in other studies was only present in Ox7, which was from part of the CORGI study that was enriched for that phenotype. Overall, our data suggest that the phenotype associated with *RNF43* is variable, can closely resemble sporadic CRC rather than polyposis cases and, as previously shown,^18^ shows incomplete penetrance. Molecular phenocopies may also occur, in which CRCs develop in mutation carriers without the inactivation of RNF43.

In general, for many TSGs that are gatekeepers^36^ and drivers in both the germ line and soma (eg*, TP53, APC, VHL, NF1, PTEN* and *SMAD4*), there is overlap between the pathogenic alleles in each context.^37^,^39^ This also appears to be the case for *RNF43* (table 1; figures 1, 4 and 5). The somatic mutation data suggested that LoF mutations throughout most of the *RNF43* gene are pathogenic, at least as far as codon 659. We were therefore surprised to find so many ‘control’ individuals without colorectal tumours who carried germline *RNF43* LoF variants, even allowing for the young age of some of these individuals. For example, given that somatic *RNF43* codon 330 nonsense mutations are recurrent in sporadic cancers and can be accompanied by LOH (https://cancer.sanger.ac.uk/cosmic/mutation/overview?id=171751745), we expected that the CRCs developed by two carriers of germline p.Arg330Ter would show second hits at *RNF43* despite their late/average age at presentation. That the cancers were both essentially phenocopies seemed less strange when we found that unaffected controls could also carry this germline mutation. It is not currently possible to measure germline variant-specific risks for *RNF43* with precision, but the somatic mutation data strongly suggest that certain germline variants not to date associated with CRC or polyps (including several at or near codon 659) are indeed intrinsically pathogenic at some level, perhaps in an appropriate setting (figure 5). We presume that lack of a ‘second hit’ or an unfavourable microenvironment means that a sufficient selective advantage does not occur. A more nuanced explanation, consistent with our germline and somatic data, encompasses the possibility that mutations earlier in the gene have stronger effects on RNF43 function than those later in the gene. It has been suggested that hypermutant cancers, including MSI+tumours, can tolerate suboptimal (but still pathogenic) driver mutations, because they can readily acquire additional mutations, such as *ZNRF3, AXIN2* and *SFRP4* here, that provide compensatory selective advantages.^40^ Currently, while we have no evidence that C-terminal *RNF43* mutations confer a raised risk of CRC, defining a clear cut-off location for mutation pathogenicity is challenging, given the small number of *RNF43* mutation carriers currently available and our evidence that somatic mutations within the C-terminal *RNF43* regions (eg, codon 659) are bona fide drivers. Nevertheless, it remains possible that pathogenic germline mutations must remove or disrupt the RING finger domain of RNF43 (approximately codons 272–313; figure 1), thus conferring a much higher CRC risk than mutations distal to this site.

Our collated data are consistent with the following model that is developing within the field (table 4). The Wnt ligand-dependent nature of *RNF43* mutations, whether germline or somatic, requires a permissive microenvironment. This is not generally present in ‘normal’ colorectal crypts with intact homoeostatic mechanisms, and hence a pre-existing precursor lesion—perhaps a HPP—is required for *RNF43* mutations to abrogate control of Wnt ligand signalling. If HPP development has a genetic basis, this could effectively lead to epistasis of the germline *RNF43* variant. Adenomatous polyps are implausible precursors, because they generally already have ligand-independent Wnt activation through *APC* mutations. In support of this model, somatic mutation and LOH of *RNF43* are frequent in serrated and filiform adenomas, but not in HPPs.^16^ In sporadic tumours, should that polyp, most likely a serrated (but non-dysplastic) lesion with a *BRAF* mutation, have already acquired defective mismatch repair, there would be a greatly enhanced tendency for frameshift mutations to occur at short repeats within *RNF43*, mostly at codons 659 and 117. The *RNF43* frameshift mutations would then lead to activated Wnt signalling, dysplasia and, in some cases, CRC. These same specific *RNF43* mutations would be uncommon in normal crypts with intact mismatch repair, and hence mutations in the *APC* gene predominate in conventional adenomas owing to the gene’s size and the mutations’ lack of dependency on Wnt ligands.

**Table 4:** Postulated model of the role of *RNF43* in serrated colorectal tumourigenesis

Component of the model	Evidence in support, where available
RNF43-associated polyposis (RAP) tumours do not initially arise through second hits at RNF43. RNF43 mutations need an existing polyp to have an effect RNF43 causes dysplasia	Normal crypt homeostasis prevents ligand-dependent Wnt activating mutations from having a selective advantage Wnt activation is associated with dysplasia Polyps in cases with germline RNF43 can be simple HPPs (eg, Yan 2020) RNF43 second hits are often found in sessile serrated adenomas
Serrated lesions (HPPs) are precursors	RNF43 polyps do have BRAF or KRAS mutations, like HPPs Precursors will not be an adenoma because they have mutated APC, hence are already Wnt active and ligand-independent, so what would RNF43 add?
Polyp formation may affect penetrance	Could be totally independent of RNF43 status Note some Ox7 non-carriers have serrated lesions Our R132X and R330X cases presented with no polyps (as did one or two others from the literature)
No MSI or CIMP needed	RAP tumours are generally MSS. MSI seems to pre-date RNF43 (or APC) in most sporadic CRCs though. But it is clearly not an obligatory component of the RNF43 pathway to CRC, as sporadic CRCs demonstrate.
N-terminal germline mutations are more pathogenic and have higher germline penetrance.	Our data testing location of germline mutations in RAP cases Our data on non-penetrance/controls Uncertain status of codon 330 germline RNF43 mutations (maybe these alleles leave RING domain mostly intact, so this is where mutations start to be a little weaker than N-terminal changes) 659 (and presumably other nearby mutations) are pathogenic, Cancer risk of some mutations near the end of the protein may be quite low,
659fs mutations are pathogenic, but the defect is likely to be weaker than that of more N-terminal mutations.	Foisted on polyps by MSI (precedent from other genes, eg, APC 1554fs in MSI+CRCs, KRAS A146T in POLE-mutant CRCs) mRNA and protein stable Supported by genetic and specific functional assays with native protein ZNRF3 mutations positively correlated with RNF43 – suggesting polygenic model The microenvironment is permissive for ligand-dependent Wnt activating changes and the codon 659 short tandem DNA repeat is more mutable than, say, the APC codon 1554 repeat.
RNF43 mutations are not more selectively advantageous than APC or CTNNB1 mutations in dysplastic serrated lesions.	

CIMP, CpG island methylatior phenotype; CRC, colorectal cancer; HPP, hyperplastic polyp; MSI, microsatellite instability/mismatch repair deficiency; RAP, RNF43-associated polyposis.

In the setting of *germline RNF43* mutations, the same model would broadly apply. Disease penetrance would be reduced in carriers who have a lower propensity to form HPPs, and correspondingly increased in polyp formers. In support of this, family Ox7, which appears to show high-penetrance inheritance of CRC and polyps, includes some affected individuals who do not carry the germline *RNF43* mutation, but do develop polyps with serrated morphology. It is very plausible (and entirely understandable) that all *RNF43* families previously reported in the literature have been subject to selection bias for a highly penetrant phenotype and hence a tendency to form HPPs. However, our data suggest that this strategy has obscured the range of *RNF43*-associated phenotypes, including non-penetrance.

Our findings enhance the limited evidence that *RNF43* is a colorectal tumour predisposition gene. Overall, germline *RNF43* mutations appear to have moderate penetrance, although this may mask considerable variation in individual risk (eg, according to individual polyp-forming tendencies or position of the germline mutation in the RNF43 protein). Taking into account other factors—including variable phenotypes, the existence of molecular phenocopies that can only be identified by cancer analysis, frequent absence of family history and the hypothetical risks of other cancers—the criteria for *RNF43* genetic testing are hard to establish and cancer prevention measures are hard to optimise. While formal cost-benefit analysis would be highly desirable and challenging, given the potential benefit for individuals, it currently remains reasonable to include *RNF43* in routine genetic testing panels for individuals with multiple polyps or familial CRC, but this will miss a considerable proportion of gene carriers with an unremarkable CRC phenotype, as indeed may well be the case for other CRC predisposition genes, such as *MSH6* and *PMS2*. Until such time as all cancer cases are screened for on a large panel of predisposition genes, there may be no solution to this issue. Our data do, however, suggest that once a germline *RNF43* variant has been identified, a pragmatic screening approach is required, for example managing patients individually by regular colonoscopy, tailored to the position of the germline variant, polyp formation and progression over time.

## Methods

Details of DNA and RNA extraction and sequencing methods used are provided in online supplemental methods, as are details of methylation analyses and how variants were annotated and scored for pathogenicity.

### Clinicopathological data

Histopathology reports and samples were provided by collaborating hospitals. 5 µm sections were stained with H&E and reviewed by two specialist colorectal pathologists (VHK, LMW) who scored the polyps according to WHO polyp guidelines to generate a consensus classification. Polyps were also collected at endoscopy, flash frozen and stored in liquid nitrogen.

### Sets of patients with CRC and/or polyps

Following the initial set of 40 CORGI individuals screened by WGS, we analysed *RNF43* germline mutations in four additional datasets of patients with CRC or colorectal polyp(s): (1) 672 from the CORGI study and collaborating studies; (2) 346 from the 100kGP^41^ Rare Disease domain (60 with familial colorectal cancer and 286 with multiple bowel polyps; (3) 3292 with CRC from the UK 100kGP Cancer Domain (whose CRCs had also undergone WGS in almost all cases); (4) 45 473 individuals with CRC, adenomas or other colorectal polyps from the UKB, including 8329 with CRC. For 100kGP, cancer registry and supporting diagnostic databases with the Research Environment were used to identify cases. For the UKB cases, main International Classification of Disease (ICD)-9 or ICD-10 coded diagnoses (field 41202) indicating colonic or rectal polyps or adenomas and colon and rectal cancer were used to identify cases with exome or genome sequencing data (http://www.ukbiobank.ac.uk/); .^10^ For UKB, some individuals with self-reported colorectal tumours (CRC or polyp) without an accompanying no ICS-9 or ICD-10 code were excluded from these cases.

### Patient data sets for association analyses

100kGP and CORGI cases (figure 4) were combined for analyses, since WGS was not performed on CORGI controls. For the association studies, we retained only a single related individual (to third degree, kinship>0.044194) for each family, preferentially keeping a CRC case over a polyp case and a polyp case over a control. We then filtered out individuals of other than European descent (defined using first PC<0.99 European for 100kGP and using principal components analysis of the genotypes (field 22006) for UKB to select Caucasian participants). For the CRC and/or polyp(s) phenotype, 3484 CORGI/100kGP individuals and 44 995 UKB individuals remained as cases; for the CRC phenotype, 3036 CORGI/100kGP individuals and 8275 UKB individuals remained as cases.

### Control data sets for association analyses

We had previously identified a set of individuals (median age 43, IQR 17) within the Rare Diseases domain of the 100kGP for use as a control set for studies to identify cancer predisposition genes and alleles. We trained a random forest model to predict continent-level ancestry (American, African, European, East Asian and South Asian) using 63 523 high confidence single nucleotide polymorphism genotypes (minor allele frequency>5 %) from the 1000 Genomes Project. Samples with European ancestry were selected by requiring the predicted probability of being European to be greater than 0.99. We also required that selected controls had no blood relative (to third degree, kinship >0.044194) within the cancer domain or another part of the 100kGP. While the completeness of the available 100kGP data could not be guaranteed, the controls selected had no reported personal or family history (1^o^ relative) of tumours, or of another disease that is a known or putative cancer risk factor (eg, type II diabetes mellitus) or could be caused by germline mutations in a somatic cancer driver gene (eg, *ARID1A*). When two controls were related, we removed the one listed first, resulting in 14 432 controls. For UK Biobank, we selected individuals in the OQFE final exome release (PLINK version) who had no ICD10 or ICD9 codes for any cancer, no ‘occurrence of cancer code’ (field 40009), no first degree relative with bowel, breast, lung or prostate cancer, no family history of malignant neoplasm of digestive organs (Z80.0). We also required that they did not answer ‘do not know’ regarding illnesses of their first-degree relatives for the group of illnesses including common cancers (group 2). As per the 100kGP selection, we only included those with European/Caucasian ancestry (based on PCs (field 22006)) who were unrelated to other controls or cases to third degree, resulting in 128 624 controls.

### Cases and controls for assessment of the effects of RNF43 mutation location on phenotype

Cases and controls were identified as per the association analyses, but ancestry and relatedness exclusion criteria were not applied. Instead, controls had to be at least 50 years of age to allow a reasonable period of time for polyps and/or CRCs to develop.

### Homozygous somatic RNF43 mutations

Somatic mutations in *RNF43* and other CRC driver genes were derived from Cornish *et al.*^25^ To distinguish between homozygous and heterozygous somatic *RNF43* mutations (in the absence of LOH), zygosity was assessed systematically across all mutations. By far the most common somatic mutation involved was the *RNF43* codon 659 frameshift, which was almost always present in MSI+cancers on a disomic chromosome 17 background. We used tumour purity estimates from cCube^42^ to calculate the expected numbers of wildtype and variant reads under competing mutational states of heterozygosity or homozygosity (where the latter was caused by two independent mutations at the same site, since LOH was identified from copy number data). We then classified cancers into three groups based on wildtype and mutant read counts: heterozygous mutant, if observed mutants were over-counted by <15% compared with those expected under heterozygosity; homozygous mutant, if observed mutants were undercounted by <15% compared with those expected under homozygosity or ‘uncertain’, where read counts fell between the limits for heterozygous or homozygous calls or the limits were overlapping (only the case for very low purity tumours). In downstream analyses, as specified in the results section, the ‘uncertain’ category was either included in a group with the heterozygous cancers, where this produced a conservative assessment or excluded. Methods used to call microsatellite instability, copy number changes and LOH in 100kGP tumours are detailed in the supplementary methods.

### Statistical analysis and data visualisation

Pedigree drawing was performed using Clinical pedigree (CJC Pedigree Software). Lollipop plots, oncoplots and MAF summaries were generated using the R statistics package ‘maftools’. Fisher’s exact tests were performed using STATA V.11.

## Supplementary material

10.1136/gutjnl-2025-337030 online supplemental file 1

10.1136/gutjnl-2025-337030 online supplemental file 2

## Data Availability

Data are available upon reasonable request.
