# Supplementary material for: Comparison between germline and somatic loss-of-function RNF43 mutations reveals different genotype-phenotype associations and provides insights into the genetic mechanisms of colorectal tumourigenesis
Source: Gut. 2025 Dec 24;75(7):e337030. doi: 10.1136/gutjnl-2025-337030 (PMC13311953; doi:10.1136/gutjnl-2025-337030)
Supplement: online supplemental file 1 [file gutjnl-75-7-s001.docx]

**SUPPLEMENTARY DATA**

| Supplementary Items | |
| --- | --- |
| 1 | Review of cases and families previously reported to carry germline *RNF43* mutations |
| 2 | Review of studies examining the pathogenicity of the 659fs somatic *RNF43* mutation |
| 3 | Consortium membership |
| Supplementary Tables | |
| 1 | Germline *RNF43* mutations reported in families with serrated polyposis |
| 2 | Information on the phenotypes reported in Family Ox7 |
| 3 | Combined database of polyps from 10 individuals in Family Ox7 from whom we received pathology reports and/or histology for review |
| 4 | Tumour-derived RNA and DNA samples from Ox7 that were available for analysis |
| 5 | Methylation beta values at CpGs annotated to *RNF43* in four polyps from Ox7:5:16 and a blood sample from the same patient |
| 6 | Targets of the single molecule molecular inversion probe (smMIP) panel |
| 7 | Primers used to assess MSI |
| 8 | Mean beta values across all CpG islands annotated to genes commonly used to assign CpG Island Methylator Phenotype status |
| 9 | Forty-three Wnt pathway genes included in the differential expression analysis presented in Supplementary Figure 3 |
| 10 | Phenotypes of individuals recruited to the CORGI study who underwent whole genome sequencing |
| 11 | Phenotypes of individuals recruited to the CORGI study or provided by collaborators who underwent whole exome sequencing |
| 12 | Distribution of 659fs genotypes deviates from that expected under no selection |
| 13 | Co-occurrence of 659fs/+ and 659fs/659s genotypes with pathogenic *APC* or *CTNNB1* alleles differs from that expected under no selection of the 659fs mutation |
| 14 | Non-659fs mutations tend to occur with 659fs and other (non-659fs) *RNF43* mutations randomly when creating pathogenic genotypes |
| 15 | Search for unsuspected Wnt pathway or other driver genes in *RNF43* 659fs*-*mutant CRCs |
| Supplementary Figures | |
| 1 | Representative images of lesion types from Ox7 at three magnifications. |
| 2 | Somatic *RNF43, BRAF* and *KRAS* mutations detected in polyps from one individual of family Ox7 |
| 3 | Differential gene expression analysis of genes in the WNT pathway in four polyps from Ox7 and 53 sporadic polyps collected by the S:CORT study |
| 4 | Box plots showing the expression of negative regulators of the Wnt pathway in polyps from Ox7 and 54 sporadic polyps collected by the S:CORT study |
| 5 | CORGI, 100kGP and UK Biobank patients and tumours |
| 6 | Breakdown of *RNF43-*mutant sporadic CRCs according to MSI and Wnt driver status |
| 7 | Nonsense-mediated decay assessment of mutant *RNF43* transcripts across 17 sporadic CRCs |
| 8 | *AXIN2* mRNA expression in different sub-groups of CRCs with *RNF43* and other Wnt driver genotypes |
| 9 | Pairwise association between Wnt driver mutations by gene in MSI+ CRCs |
| Supplementary Methods | |
| Supplementary References | |

**Supplementary Item 1. Review of cases and families previously reported to carry germline *RNF43* mutations.**

Loss of function *RNF43* mutations have previously been reported in 11 families (10,15–18,61), mainly selected for their multiple polyp phenotype (**Supplementary Table 1**).

1,2. In 2014, Gala et al [1] reported germline *RNF43* p.Arg113Ter mutations in two unrelated individuals with serrated polyposis syndrome (SPS). Very limited additional molecular analysis related to tumours was reported.

3. In 2015, Taupin et al [2] found an *RNF43* p.Arg132Ter mutation in members of a SPS kindred. The proband had >50 large serrated colorectal polyps, one of which progressed to an MSI- CRC, a sibling had multiple serrated polyps, another had a single adenoma, and their mother had pancreatic cancer.

4. Yan et al [3] reported a single SPS kindred with an *RNF43* splice site mutation (c.953-1, G>A) that caused a frameshift at codon 318 and a truncated protein; tumours in two gene carriers were predominantly multiple serrated adenomas, with accompanying hyperplastic polyps and classical adenomas, but a further carrier had an MSI-negative CRC with just one classical adenoma and a hyperplastic polyp. Almost all polyps showed evidence of LOH or truncating second hits at *RNF43.* However*,* the method used to assess LOH appeared to be visual inspection of cDNA sequencing traces. This is prone to confounding by transcript stability and variable allele-specific expression of unknown causes, particularly given the germline splice site mutation here. Many tumours had somatic *BRAF* mutations.

5. Quintana et al [4] identified *RNF43* p.Arg132Ter in one of 96 unrelated patients with SPS. The carrier was diagnosed with CRC and >50 polyps at age 55. The CRC was CIMP+.

6. Mikaeel et al [5] recently found a germline *RNF43* splice variant (c.375+1, G>A) that caused a frameshift at codon 126 and truncated protein. The proband had two metachronous CRCs and seven polyps (two hyperplastic, thee sessile serrated and two classical adenomatous) from 50 years of age. A second hit at *RNF43* was found in the CRC*.* The phenotype in terms of polyp numbers and age of presentation was less severe than in other studies.

7. Chan et al [6] identified a family with RNF43:c.988 C > T;p.Arg330Ter. However, a pathogenic *BRCA1* variant (c.2681_2682delAA p.Lys894ThrfsTer8) was also present in 10 individuals, 7 of whom also carried the *RNF43* variant, whereas one carried the *RNF43* variant alone. Four of the *RNF43* carriers developed CRC before age 56, with two also diagnosed with polyps. Notably, the other four carriers were affected with other cancers (N=2) or remained unaffected in their 50s (N=2).

8. Our data on Ox7 are included for completeness, since this family was identified based on their multiple polyp phenotype, via the proband.

9. Recently, Brinch et al [7] reported a multi-generational family with a SPS and CRC phenotype (N=3), and a germline deletion of *RNF43* exons 4-9, starting close to codon 125. Other families without CRC or polyposis with germline *RNF43* mutations (p.Arg337Ter and p.Arg650Ter) were also reported.

**Supplementary Item 2. Review of studies examining the pathogenicity of the 659fs somatic *RNF43* mutation.**

In order to inform our germline analyses, we initially undertook a literature review of somatic *RNF43* driver mutations, particularly 659fs, which provides a way to explore whether protein-truncating mutations in the C-terminal/cytoplasmic domain of the *RNF43* gene are pathogenic . Some previous studies have claimed that 659fs mutant proteins are functionally indistinguishable from wildtype as regards expression, stability, localisation, Dvl binding, R-spondin binding, inhibition of Wnt signalling [8, 9, 10], consistent with a paucity of somatic C-terminal *RNF43* mutations in MSS cancers (**Figure 4**). However, Yu et al [11] and Fang et al [12] reported that endogenous 659fs and similar C-terminal truncating mutations are essentially LoF alleles, the impacts of which were not detectable by the transient transfection *in vitro* assays used in most previous studies. Some of these studies provided evidence that the functional deficiency of 659fs changes is not complete, which might well explain the apparently discordant data.

**Supplementary Item 3. Consortium membership**

**S:CORT Consortium members:**

Andrew Blake^1^, Enric Domingo^1^, Viktor H. Koelzer^1^, Simon J. Leedham^2^, Timothy S. Maughan^1^, Susan D. Richman^3^, Philip D. Dunne^4^, Mark Lawler^4^ & Keara L. Redmond^4^

^1^Department of Oncology, University of Oxford, Oxford, Oxfordshire, UK, ^2^Centre for Human Genetics, University of Oxford, Oxford, UK, ^3^Leeds Institute of Medical Research, University of Leeds, Leeds, UK, ^4^The Patrick G Johnston Centre for Cancer Research, Queen’s University Belfast, Belfast, UK

**UK Colorectal Cancer Genomics Consortium:**

Alex J. Cornish^1,26^, Andreas J. Gruber^2,3,26^, Ben Kinnersley^1,4,26^, Daniel Chubb^1,26^, Anna Frangou^5,6,26^, Giulio Caravagna^7,8,26^, Boris Noyvert^9,26^, Eszter Lakatos^8,10,26^, Henry M. Wood^11,26^, Steve Thorn^12,26^, Richard Culliford^1,26^, Claudia Arnedo-Pac^13,14,15^, Jacob Househam^8^, William Cross^8,16^, Amit Sud^1^, Philip Law^1^, Maire Ni Leathlobhair^17^, Aliah Hawari^3^, Connor Woolley^12^, Kitty Sherwood^12,18^, Nathalie Feeley^12,18^, Güler Gül^18^, Juan Fernandez-Tajes^12^, Luis Zapata^8^, Ludmil B. Alexandrov^19,20,21^, Nirupa Murugaesu^22^, Alona Sosinsky^22^, Jonathan Mitchell^22^, Nuria Lopez-Bigas^13,14,15^, Philip Quirke^11,27^, David N. Church^23,24,27^, Ian P. M. Tomlinson^12,27^, Andrea Sottoriva^8,25,27^, Trevor A. Graham^8,27^,David C. Wedge^3,27^ & Richard S. Houlston^1,27^

^1^Division of Genetics and Epidemiology, Institute of Cancer Research, London, UK. ^2^Department of Biology, University of Konstanz, Konstanz, Germany. ^3^Manchester Cancer Research Centre, Division of Cancer Sciences, University of Manchester, Manchester, UK. ^4^University College London Cancer Institute, London, UK. ^5^Big Data Institute, Nuffield Department of Medicine, University of Oxford, Oxford, UK. ^6^Max Planck Institute for Molecular Cell Biology and Genetics, Dresden, Germany. ^7^Department of Mathematics and Geosciences, University of Trieste, Trieste, Italy. ^8^Centre for Evolution and Cancer, Institute of Cancer Research, London, UK. ^9^Cancer Research UK Centre and Centre for Computational Biology, Institute of Cancer and Genomic Sciences, University of Birmingham, Birmingham, UK ^10^Department of Mathematical Sciences, Chalmers University of Technology, Gothenburg, Sweden. ^11^Pathology and Data Analytics, Leeds Institute of Medical Research at St James’s, University of Leeds, Leeds, UK. ^12^Department of Oncology, University of Oxford, Oxford, UK. ^13^Institute for Research in Biomedicine Barcelona, The Barcelona Institute of Science and Technology, Barcelona, Spain. ^14^Centro de Investigación Biomédica en Red de Cáncer (CIBERONC), Barcelona, Spain. ^15^Institució Catalana de Recerca i Estudis Avançats (ICREA), Barcelona, Spain. ^16^Research Department of Pathology, University College London, UCL Cancer Institute, London, UK. ^17^Trinity College, Dublin, Ireland. ^18^Edinburgh Cancer Research, Institute of Genetics and Cancer, University of Edinburgh, Edinburgh, UK. ^19^Department of Cellular and Molecular Medicine, UC San Diego, La Jolla, CA, USA. ^20^Department of Bioengineering, UC San Diego, La Jolla, CA, USA. ^21^Moores Cancer Center, UC San Diego, La Jolla, CA, USA. ^22^Genomics England, William Harvey Research Institute, Queen Mary University of London, London, UK. ^23^Wellcome Centre for Human Genetics, University of Oxford, Oxford, UK. ^24^Oxford NIHR Comprehensive Biomedical Research Centre, Oxford University Hospitals NHS Foundation Trust, Oxford, UK. ^25^Computational Biology Research Centre, Human Technopole, Milan, Italy.

**The CORGI Consortium:**

Dr. Kai Ren Ong (Birmingham Women's Hospital, Birmingham), Prof. Andrew Beggs (Institute of Cancer and Genomic Sciences, University of Birmingham), Dr. Alan Donaldson (St. Michael's Hospital, Dr. Ruth Armstrong, Addenbrooke's NHS Trust, Cambridge), Dr. Carole Brewer (Royal Devon & Exeter Hospital (Heavitree)), Exeter, Prof. Jayantha Arnold (Ealing Hospital, Middlesex), Dr. Munaza Ahmed (Great Ormond Street Hospital, London), Dr. Louise Izatt (Guy's Hospital**,** London), Dr. Andrew Latchford (St Mark’s Hospital , Harrow and Division of Surgery and Cancer, Imperial College London), Dr Dorothy Halliday (Nuffield Orthopaedic Hospital, Oxford), Peter Risby (The Oxford Genomic Medicine Centre, Oxford), Dr Paul Brennan (The James Cook University Hospital, Middlesbrough), Dr. Alison Kraus (Chapel Allerton Hospital, Leeds), Dr. Julian Barwell (Leicester Royal Infirmary, Leicester), Dr. Lynn Greenhalgh (Liverpool Women's Hospital), Prof. D. Gareth Evans (University of Manchester, Manchester), Kate Green (University of Manchester, Manchester), Dr. Timothy Simmons (Institute of Genetic Medicine, International Centre for Life, Newcastle upon Tyne), Dr. Rachel Harrison (City Hospital Campus, Nottingham), Prof. Ragunath (Queen's Medical Centre Campus, Nottingham), Prof. Brian Davidson (Royal Free Hampstead NHS Trust, University Dept. of Liver Medicine & Transplantation, London), Dr. Zoe Kemp (The Royal Marsden, Sutton), Dr. Helen Hanson (St George’s University, London), Dr Katie Snape (St George’s University, London), Prof. Anneke Lucassen (Princess Anne Hospital, Southampton), Dr. Kevin J Monahan (West Middlesex University Hospital, Middlesex), Prof. Patrick Morrison (City Hospital Campus, Belfast).

**WGS500 consortium:**

Peter Donnelly^1^, John Bell^2^ , David Bentley^3^, Gil McVean^1^, Peter Ratcliffe^1^, Jenny Taylor^1,4^, Andrew Wilkie^4, 5,^ John Broxholme^1^, David Buck^1^, Jean-Baptiste Cazier^1^, Richard Cornall^1^, Lorna Gregory^1^, Julian Knight^1^, Gerton Lunter^1^, Ian Tomlinson^1, 4,^ Christopher Allan^1^, Moustafa Attar^1^, Angie Green^1^, Sean Humphray^3,^ Zoya Kingsbury^3^, Sarah Lamble^1^, Lorne Lonie^1^, Alistair Pagnamenta^1^, Paolo Piazza^1^, Guadelupe Polanco^1^, Amy Trebes^1^,Richard Copley^1^, Simon Fiddy^1^, Russell Grocock^3^, Edouard Hatton^1^, Chris Holmes^1^, Linda Hughes^1^, Peter Humburg^1^, Alexander Kanapin^1^, Stefano Lise^1^, Hilary Martin^1^, Lisa Murray^3^ , Davis McCarthy^1^, Andy Rimmer^1^, Natasha Sahgal^1^, Ben Wright^1^, Chris Yau^6^

^1^ The Wellcome Trust Centre for Human Genetics, Roosevelt Drive, Oxford, OX3 7BN, UK. ^2^ Office of the Regius Professor of Medicine, Richard Doll Building, Roosevelt Drive, Oxford, OX3 7LF, UK ^3^ Illumina Cambridge Ltd., Chesterford Research Park, Little Chesterford, Essex, CB10 1XL, UK ^4^ NIHR Oxford Biomedical Research Centre, Oxford, UK.^5^ Weatherall Inst of Molecular Medicine, University of Oxford; John Radcliffe Hospital, Headington, Oxford OX3 9DS, UK^6^ Imperial College London, South Kensington Campus, London, SW7 2AZ. UK

**Supplementary Table 1:** **Germline *RNF43* mutations reported in families with serrated polyposis syndrome (see Supplementary Item 1)**

| Family | Individual | Germline mutation | Phenotype | MSI | CIMP  CRC | CIMP  polyps | KRAS  CRC | KRAS  polyps | BRAF  CRC | BRAF  polyps | RNF43  2^nd^ hit | RNF43  LOH |
| --- | --- | --- | --- | --- | --- | --- | --- | --- | --- | --- | --- | --- |
| 1 | 1 | c.337C>T p.Arg113Ter | SPS (>30 SSLs), age 51 |  |  |  |  |  |  |  |  | Not assessed |
| 2 | 1 | c.337C>T p.Arg113Ter | CLL (age 42) and SPS (7 SSLs), age 52 |  |  |  |  |  |  |  |  | Not assessed |
| 3 | 1.1 | c.394C>T p.Arg132Ter | multiple polyps and CRC, age 23 | - |  |  | WT |  |  |  |  | Not assessed |
| 3 | 1.2 | c.394C>T p.Arg132Ter | >80 serrated polyps, age 27 |  |  |  |  |  |  |  |  | Not assessed |
| 4 | 1.1 | c.953-1G>A p.Glu318fs | >100 polyps (SSL, HP, adenomas), age 65 |  | - | 61.5% + |  | 19% mutant |  | 62% mutant |  | 87.5% of SSLs, TSAs and HPs showed LOH, 80% of TVA and 100% of cancers showed LOH (see note below) |
| 4 | 1.2 | c.953-1G>A p.Glu318fs | >20 polyps (SSL HP), age 64 |  |  |  |  |  |  |  |  |  |
| 4 | 1.3 | c.953-1G>A p.Glu318fs | 1 HP (53), rectal cancer & 1 adenoma, age 49 |  |  |  |  |  |  |  |  |  |
| 4 | 1.4 | c.953-1G>A p.Glu318fs | 2 SSLs and 1 adenoma (age 37) |  |  |  |  |  |  |  |  |  |
| 4 | 1.5 | c.953-1G>A p.Glu318fs | 1 SSL and 2 HP (age 35) |  |  |  |  |  |  |  |  |  |
| 4 | 1.6 | c.953-1G>A p.Glu318fs | normal colonoscopy age 44; no more investigation to age 60 |  |  |  |  |  |  |  |  |  |
| 5 | 1 | c.394C>T p.Arg132Ter | CRC, age 55 and >50 serrated polyps |  | + |  | WT |  | WT |  | c.2309 -1G>A CRC |  |
| 6 | III:1 | c.375+1G>A p.A126Ifs*50 | CRC and colorectal polyp, age 50 | - |  |  |  |  | V600E |  | None |  |
| 6 | II:2 | c.375+1G>A p.A126Ifs*50 | CRC, age 65; colorectal polyps, age 65, 66, 68 | - |  |  |  |  | V600E |  | p.Arg145Ter CRC |  |
| 7 | 001 | c.988C>T p.Arg330Ter | Caecal ca., age 53; peritoneal ca., age 62; ovarian ca., age 63 & many small metaplastic polyps | - |  |  |  |  |  |  |  | Not assessed |
| 7 | 014 | c.988C>T p.Arg330Ter | CRC, age 56; prostate cancer, age 71 | - | - |  | WT codon 12 & 13 |  | WT |  |  | Not assessed |
| 7 | 025 | c.988C>T p.Arg330Ter | Breast & ovarian cancer |  |  |  |  |  |  |  |  |  |
| 7 | 009 | c.988C>T p.Arg330Ter | CRC, age 44; no report of polyps | - | - |  | WT codon 12 & 13 |  | WT |  |  | LOH (CRC) |
| 7 | 010 | c.988C>T p.Arg330Ter | CRC in SSL age 56; multiple serrated and adenomatous polyps |  | + |  |  |  | V600E |  |  | LOH sessile serrated lesion & CRC |
| 7 | 026 | c.988C>T p.Arg330Ter | Unaffected at 58 |  |  |  |  |  |  |  |  |  |
| 7 | 027 | c.988C>T p.Arg330Ter | Other cancer (no details) |  |  |  |  |  |  |  |  |  |
| 7 | 028 | c.988C>T p.Arg330Ter | Unaffected, age likely 50s |  |  |  |  |  |  |  |  |  |
| 8 | Ox7 5.16 | c.471del p.Thr158ProfsTer6 | 19 SSLs, 3TSAs, 2 HPs | - |  |  |  |  |  |  |  | Not assessed |
| 8 | Ox7 6.1 | c.471del p.Thr158ProfsTer6 | 30 adenomas including 19 SSLs, 2 TSAs, 1 TA, 1 HP | - | - |  |  |  |  |  |  | 1/7 with LOH |
| 8 | Ox7 6.2 | c.471del p.Thr158ProfsTer6 | CRC, age 46; multiple serrated adenomas | - |  |  |  |  |  |  |  | Not assessed |
| 8 | Ox7 6.3 | c.471del p.Thr158ProfsTer6 | 14 polyps, no morphology available | - |  |  |  |  |  |  |  | Not assessed |
| 9 | I.3 | Exon4-9 deletion | CRC proximal colon ,age 70; 3 TAs & 13 SSPs | - |  |  |  |  |  |  |  |  |
| 9 | I.4 | Exon4-9 deletion | CRC, age 65; polyps unknown | Loss ofMLH1 & PMS2 |  |  |  |  |  |  |  |  |
| 9 | II.2 | Exon4-9 deletion | 6 TAs & 44 SSPs (age 44-56) | - |  |  |  |  |  |  |  |  |
| 9 | III.1 | Exon4-9 deletion | Polyp status unknown |  |  |  |  |  |  |  |  |  |
| 9 | III.2 | Exon4-9 deletion | Polyp status unknown |  |  |  |  |  |  |  |  |  |
| 10 | II | p.Arg650Ter | Gastric ca. (age 61), 1 TA (age 57) | Loss of MSH2 & MSH6 |  |  |  |  |  |  |  |  |
| 11 | III | p.Arg650Ter | 1 TA |  |  |  |  |  |  |  |  |  |
| 12 | IV | p.Arg337Ter | No polyps @ multiple endoscopies |  |  |  |  |  |  |  |  |  |

**Supplementary Table 2****: Information on the phenotypes reported in Family Ox7**

We performed whole-genome sequencing (WGS) of constitutional DNA from family member 5.16, who by age 78 had developed CRC and 31 polyps, the majority of which were of sessile serrated morphology (**Supplementary Tables 1 & 2**). Family member 6.1 (30 polyps by age 52) was subsequently sequenced. Although the family’s clinical features suggested Lynch syndrome, no pathogenic germline mismatch repair (MMR) gene variants had been reported by diagnostic genetics laboratories or were identified by our analyses. We found five high confidence, heterozygous LoF variants in other genes that were shared by 5.16 and 6.1 and were very rare in other data sets. Four variants were in genes with no evidence of a role in cancer predisposition:

(i) *QPCT* p.Arg133Ter

(ii) *MAN2B2* p.Gln405Ter

(iii) *AP3B2* p.Trp808Ter

(iv) *KLHDC7B* p.Lys822SerfsTer65.

One variant was a frameshift deletion in *RNF43* (ENST00000407977.2:c.471del; ENSP00000385328.2:p.Thr158ProfsTer6; **Figure 1C**). This variant, which was confirmed in the Regional Clinical Genetics Laboratory [13], was present once in gnomAD v3.1.2 samples (total allele count=64,792, allele frequency =1.54 x10^-5^; **Figure 1A**), but was otherwise absent from the literature (**Figure 1B**) and public databases, including the control and biobank samples in gnomAD v3.1.2 (allele count=6,846). The variant was present in Ox7 individuals 5.16, 6.1, 6.2, 6.3 and by inference, 5.19 (**Figure 2**). All these gene carriers had developed >10 polyps and 5.19 and 6.2 had developed CRC. On review by VK and LMW, polyp morphology was predominantly reported as sessile serrated lesions (SSLs), hyperplastic polyps (HPPs) and tubular adenomas (TAs) (**Supplementary Table 3**). Non-gene carriers 6.4, 6.5 and 6.9, who had been undergoing yearly screening colonoscopy owing to their family history, had also developed polyps, respectively from their records <5 HPPs, one 1cm diameter SSL, and 3 TAs (**Supplementary Table 2**). Despite the exclusion of Lynch syndrome by several clinical laboratories involved in the family’s care, we wondered whether a second Mendelian CRC gene was present in some family members and hence performed WGS on individual 6.5, given the large size of the SSL removed.

| Individual ID | Sex | Phenotypes | Age at diagnosis | Germline genetic analysis |
| --- | --- | --- | --- | --- |
| 1.1 | M |  |  |  |
| 1.2 | F |  |  |  |
| 2.1 | M |  |  |  |
| 2.2 | F |  |  |  |
| 2.3 | M |  |  |  |
| 2.4 | F |  |  |  |
| 2.5 | M |  |  |  |
| 2.6 | F |  |  |  |
| 3.1 | M |  |  |  |
| 3.2 | F | CRC | 65 |  |
| 3.3 | M |  |  |  |
| 3.4 | F |  |  |  |
| 3.5 | M |  |  |  |
| 3.6 | F |  |  |  |
| 3.7 | M |  |  |  |
| 3.8 | F |  |  |  |
| 3.9 | M |  |  |  |
| 3.10 | F |  |  |  |
| 3.11 | M |  |  |  |
| 3.12 | F |  |  |  |
| 3.13 | M |  |  |  |
| 3.14 | F |  |  |  |
| 3.15 | M |  |  |  |
| 3.16 | F |  |  |  |
| 4.1 | F |  |  |  |
| 4.2 | M |  |  |  |
| 4.3 | F |  |  |  |
| 4.4 | M |  |  |  |
| 4.5 | M |  |  |  |
| 4.6 | F |  |  |  |
| 4.7 | M |  |  |  |
| 4.8 | F |  |  |  |
| 4.9 | M | Prostate cancer | 82 |  |
| 4.10 | F | CRC | 48 |  |
| 4.11 | M |  |  |  |
| 4.12 | F |  |  |  |
| 4.13 | M | CRC | 54 |  |
| 4.14 | F |  |  |  |
| 4.15 | M | Bladder cancer | 55 |  |
| 4.16 | M | Polyps |  |  |
| 4.17 | F |  |  |  |
| 4.18 | M | Polyps |  |  |
| 4.19 | F |  |  |  |
| 4.20 | M | CRC |  |  |
| 4.21 | F | CRC and endometrial cancer |  |  |
| 4.22 | M |  |  |  |
| 4.23 | F | Other cancer not specified |  |  |
| 4.24 | F |  |  |  |
| 4.25 | F | CRC |  |  |
| 4.26 | M | CRC |  |  |
| 4.27 | F |  |  |  |
| 4.28 | M | CRC |  |  |
| 4.29 | F | CRC |  |  |
| 4.30 | F | CRC |  |  |
| 4.31 | M |  |  |  |
| 4.32 | M | CRC |  |  |
| 4.33 | F |  |  |  |
| 5.1 | M |  |  |  |
| 5.2 | M |  |  |  |
| 5.3 | M |  |  |  |
| 5.4 | M |  |  |  |
| 5.5 | M |  |  |  |
| 5.6 | F |  |  |  |
| 5.7 | F |  |  |  |
| 5.8 | F | 45 hyperplastic polyps |  |  |
| 5.9 | F |  |  |  |
| 5.10 | F |  |  |  |
| 5.11 | F |  |  |  |
| 5.12 | M | CRC | 60 |  |
| 5.13 | M |  |  |  |
| 5.14 | M |  |  |  |
| 5.15 | M |  |  |  |
| 5.16 | M | Basal cell carcinoma, prolactinoma, keratoacanthoma and multiple adenomas of various morphologies | 43-78 | WGS |
| 5.17 | F |  |  |  |
| 5.18 | F |  |  |  |
| 5.19 | M | CRC and adenomas | 39 |  |
| 5.20 | F |  |  |  |
| 5.21 | M |  |  |  |
| 5.22 | F | 1 hyperplastic polyp | 59 |  |
| 5.23 | F | 10-15 hyperplastic polyps |  |  |
| 5.24 | F |  |  |  |
| 5.25 | M | CRC |  |  |
| 5.26 | F | CRC |  |  |
| 5.27 | F | CRC |  |  |
| 5.28 | F | 1 hyperplastic polyp |  |  |
| 5.29 | M | 1 hyperplastic polyp |  |  |
| 5.30 | M |  |  |  |
| 5.31 | M | Multiple hyperplastic polyps |  |  |
| 5.32 | M |  |  |  |
| 5.33 | F |  |  |  |
| 5.34 | F |  |  |  |
| 5.35 | M | Multiple hyperplastic polyps |  |  |
| 5.36 | F | Multiple hyperplastic polyps |  |  |
| 5.37 | F | Multiple hyperplastic polyps |  |  |
| 5.38 | M | CRC | 55 |  |
| 5.39 | F | Multiple hyperplastic polyps |  |  |
| 5.40 | F | Multiple hyperplastic polyps |  |  |
| 5.41 | F | CRC |  |  |
| 5.42 | M | CRC |  |  |
| 5.43 | F |  |  |  |
| 5.44 | M |  |  |  |
| 5.45 | M | CRC |  |  |
| 6.1 | F | 30 adenomas including SSLs, TSAs, hyperplastic polyps & 1 TA | 29-52 | WGS |
| 6.2 | F | CRC and 4 serrated adenomas | polyps since 34, CRC at 46 | genotyping array |
| 6.3 | M | 14 polyps, type not specified |  | genotyping array |
| 6.4 | F | <5 hyperplastic polyps |  | genotyping array |
| 6.5 | F | 1 SSL (>1 cm) |  | WGS |
| 6.6 | M |  |  |  |
| 6.7 | M |  |  |  |
| 6.8 | F |  |  |  |
| 6.9 | M | 3 tubular adenomas | 39 | targeted Sanger sequencing |
| 6.10 | M |  |  |  |
| 6.11 | F |  |  |  |
| 6.12 | M | Polyps not specified |  |  |

**Supplementary Table 3:** **Combined database of polyps from 10 individuals in Family Ox7 from whom we received pathology reports and/or histology for review**

| **Pedigree**  **ID** | **Polyp**  **ID** | **Pathology from report** | **Independent review of morphology** | **Organ** | **Location** | **Size, mm** |
| --- | --- | --- | --- | --- | --- | --- |
| 5.16 | 1 | Sclerosed haemangioma | Not available | Liver | Segment IV |  |
| 5.16 | 2 | Tubular adenoma | Not available | Colon | Caecum | 10 |
| 5.16 | 3 | Hyperplastic polyp | Not available | Colon | Proximal Ascending | 12 |
| 5.16 | 4 | Serrated polyp (partially serrated adenoma/hyperplastic) | Not available | Colon | Rectum | 3 |
| 5.16 | 5 | Gastritis | Not available | Stomach |  |  |
| 5.16 | 6 | Traditional serrated adenoma | SSL with serrated dysplasia (low grade) | Colon | Rectum | 3 |
| 5.16 | 8 | Tubular adenoma | SSL with intestinal dysplasia (low grade) | Colon | Ascending | 5 |
| 5.16 | 9 | Sessile serrated | SSL | Colon | Ascending | 7 |
| 5.16 | 10 | Tubular adenoma | Tubular adenoma with low grade dysplasia | Colon | Ascending | 4 |
| 5.16 | 11 | Serrated polyp | SSL | Colon | Ascending | 6 |
| 5.16 | 12 | Crushed biopsies (possible hyperplastic polyps) | Crushed biopsies (possible hyperplastic polyps) | Colon | Caecum |  |
| 5.16 | 13 | Crushed biopsies (possible hyperplastic polyps) | Crushed biopsies (possible hyperplastic polyps) | Colon | Ascending |  |
| 5.16 | 14 | Hyperplastic polyp | SSL | Colon | Proximal Ascending |  |
| 5.16 | 15 | Hyperplastic polyp | SSL | Colon | Proximal Ascending |  |
| 5.16 | 16 | Hyperplastic polyp | SSL with intestinal dysplasia (low grade) | Colon | Hepatic flexure |  |
| 5.16 | 17 | Hyperplastic polyp | SSL with serrated dysplasia (low grade) | Colon | Splenic flexure |  |
| 5.16 | 18 | Hyperplastic polyp | SSL | Colon | Proximal descending |  |
| 5.16 | 19 | Hyperplastic polyp | SSL | Colon | Splenic flexure |  |
| 5.16 | 20 | Hyperplastic polyp | SSL | Colon | Transverse |  |
| 5.16 | 21 | Tubular adenoma | SSL with intestinal dysplasia (low grade) | Colon | Caecum | 1 |
| 5.16 | 22 | Normal | Not available | Duodenum |  |  |
| 5.16 | 23 | Normal | Not available | Stomach | Antrum |  |
| 5.16 | 24 | Non-specific chronic gastritis (mild) | Not available | Stomach | Gastric body |  |
| 5.16 | 25 | Chronic inflammation | Not available | Stomach | Antrum | 3 |
| 5.16 | 26 | Chronic inflammation | Not available | Stomach | Gastric body | 2 |
| 5.16 | 27 | Reactive/chemical gastritis | Not available | Stomach | Antrum |  |
| 5.16 | 28 | Chronic inflammation | Not available | Lower oesophagus |  |  |
| 5.16 | 29 | Tubular adenoma | Tubular adenoma with low grade dysplasia | Colon | Caecum |  |
| 5.16 | 30 | Hyperplastic polyp | SSL | Colon | Proximal Ascending |  |
| 5.16 | 31 | Hyperplastic polyp | SSL with intestinal dysplasia (low grade) | Colon | Proximal sigmoid |  |
| 5.16 | 32 | Hyperplastic polyp | SSL with intestinal dysplasia (low grade) | Colon | Proximal sigmoid |  |
| 5.16 | 33 | Keratoacanthoma | Not available | Skin | Left shoulder |  |
| 5.16 | 34 | Basal cell carcinoma | Not available | Skin | Left shoulder |  |
| 5.16 | 37 | Traditional serrated polyp | SSL with serrated dysplasia (low grade) | Colon | Proximal sigmoid | 3 |
| 5.16 | 38 | Sessile serrated | SSL with intestinal dysplasia (low grade) | Colon | Proximal sigmoid | 3 |
| 5.16 | 39 | Sessile serrated | SSL with intestinal dysplasia (low grade) | Colon | Splenic flexure | 3 |
| 5.16 | 40 | Traditional serrated adenoma | SSL with serrated dysplasia (low grade) | Colon | Proximal descending | 4 |
| 5.16 | 42 | Hyperplastic/serrated | SSL with serrated dysplasia (low grade) | Colon | Caecum |  |
| 5.16 | 43 | Tubular adenoma | Tubular adenoma (low grade) | Colon | Caecum | 3 |
| 5.16 | 44 | Tubular adenoma | Tubular adenoma (low grade) | Colon | Ascending | 4 |
| 5.16 | 46 | Serrated adenoma | SSL with intestinal dysplasia (low grade) | Colon | Ascending |  |
| 5.16 | 47 | Serrated adenoma | SSL with intestinal dysplasia (low grade) | Colon | Descending |  |
| 5.16 | 86 |  | SSL with intestinal dysplasia (low grade) | Colon | Ascending |  |
| 5.16 | 88 |  | SSL with serrated dysplasia (low grade) | Colon | Transverse |  |
| 5.16 | 89 |  | SSL | Colon | Descending |  |
| 5.22 |  | Cyst | Not available | Kidney | Left |  |
| 5.22 |  | Cyst | Not available | Kidney | Cortical | 4 |
| 5.22 |  | Linear polyp (hyperplastic) | Not available | Colon | Cecum | 10 |
| 5.22 |  | Sessile mucosal elevation | Not available | Colon | Proximal sigmoid | 2 |
| 5.23 |  | Polyp | Not available | Colon | caecum | 4 |
| 5.23 |  | Polyp | Not available | Colon | caecum | 3 |
| 5.23 |  | Flat polyp. Suggested as serrated adenoma. | Not available | Colon | hepatic flexure | 10 |
| 5.23 |  | Flat polyp. Suggested as serrated adenoma. | Not available | Colon | hepatic flexure | 10 |
| 5.23 |  | Flat polyp. Suggested as serrated adenoma. | Not available | Colon | hepatic flexure | 10 |
| 5.23 |  | Flat polyp | Not available | Colon | splenic flexure | 10 |
| 5.23 |  | Sessile polyps | Not available | Colon | sigmoid colon | 10 |
| 5.23 |  | Polyp | Not available |  |  |  |
| 5.23 |  | Remains of a polyp | Not available | Colon | splenic flexure | 2 |
| 5.23 |  | Small nodule/early polyp | Not available |  | sigmoid flexure | ? |
| 5.23 |  | Polyp | Not available |  | sigmoid flexure |  |
| 5.23 |  | Polyp | Not available |  | splenic flexure |  |
| 5.23 |  | Polyp | Not available |  | splenic flexure |  |
| 5.23 |  | Polyp | Not available |  | descending colon | 10 |
| 5.23 |  | Polyp | Not available |  | descending colon | 5 |
| 5.24 |  | Hyperplastic | Not available |  | descending colon | ? |
| 5.8 |  | 45 hyperplastic polyps | Not available | Colon | Rectum | 4 |
| 5.8 |  | Diverticulitis | Not available |  |  |  |
| 6.1 | 48 | Hyperplastic polyp | SSL | Colon |  | 5 |
| 6.1 | 49 | Benign Dermatofibroma | Not available | Skin | Right wrist | 4 |
| 6.1 | 54 | Serrated adenoma | TSA | Colon | Splenic flexure | 15 |
| 6.1 | 55 | Serrated adenoma | TSA | Colon | Proximal sigmoid | 9 |
| 6.1 | 56 | Sessile serrated | SSL | Colon | Caecum | 8 |
| 6.1 | 57 | Sessile serrated | SSL with serrated dysplasia (low grade) | Colon | Proximal ascending | 10 |
| 6.1 | 58 | Hyperplastic polyp | SSL | Colon | Mid transverse | 6 |
| 6.1 | 59 | Serrated adenoma | Not available | Colon | Hepatic flexure |  |
| 6.1 | 60 | Serrated adenoma | Not available | Colon |  | 4 |
| 6.1 | 61 | Normal | Not available | Colon | Ileocaecal valve |  |
| 6.1 | 62 | Traditional serrated adenoma | Not available | Colon | Mid transverse |  |
| 6.1 | 63 | Traditional serrated adenoma | Not available | Colon | Proximal sigmoid |  |
| 6.1 | 64 | Sessile serrated | SSL | Colon | Splenic flexure |  |
| 6.1 | 65 | Sessile serrated | SSL with serrated dysplasia (low grade) | Colon | Rectum |  |
| 6.1 | 66 | Sessile serrated | SSL | Colon | Mid transverse |  |
| 6.1 | 67 | Sessile serrated | SSL | Colon | Splenic flexure |  |
| 6.1 | 68 | Sessile serrated | SSL | Colon | Proximal ascending |  |
| 6.1 | 69 | Sessile serrated | SSL | Colon | Proximal ascending |  |
| 6.1 | 70 | Hyperplastic polyp | SSL | Colon | Ascending |  |
| 6.1 | 71 | Sessile serrated | SSL with serrated dysplasia (low grade) | Colon | Ascending |  |
| 6.1 | 72 | Sessile serrated | SSL | Colon | Transverse |  |
| 6.1 | 73 | Hyperplastic polyp | SSL | Colon | Proximal sigmoid |  |
| 6.1 | 74 | Hyperplastic polyp | SSL | Colon | Proximal sigmoid |  |
| 6.1 | 75 | Normal | Not available | Duodenum | 2nd part |  |
| 6.1 | 76 | Reactive gastritis | Not available | Stomach | Antrum |  |
| 6.1 | 77 | Cystic fundic gland polyp | Not available | Stomach | Middle body |  |
| 6.1 | 78 | Reactive pan gastritis | Not available | Stomach | Upper body |  |
| 6.1 | 79 | Squamous epithelium | Not available | Oesophagus | Lower |  |
| 6.1 | 80 | Hyperplastic polyp | SSL | Colon | Ascending |  |
| 6.1 | 81 | Serrated polyp | SSL | Colon | Transverse |  |
| 6.1 | 82 | Serrated polyp | SSL | Colon | Splenic flexure |  |
| 6.1 | 83 | Hyperplastic polyp | SSL | Colon | Rectum |  |
| 6.1 | 84 | Tubular adenoma | Tubular adenoma (low grade) | Colon | Low rectum |  |
| 6.1 | 85 | Hyperplastic polyp | Hyperplastic polyp | Colon | Rectum |  |
| 6.2 |  | Serrated adenoma | Not available | colon | sigmoid |  |
| 6.2 |  | Tubular adenoma no dysplasia | Not available | colon | colon at 60cm |  |
| 6.2 |  | Serrated/tubular adenoma | Not available | colon | ascending colon |  |
| 6.2 |  | Serrated/tubular adenoma | Not available | colon | ascending colon |  |
| 6.2 |  | Adenocarcinoma arising in serrated/tubular adenoma | Not available | colon | ascending colon |  |
| 6.5 |  | Sessile serrated adenoma | Not available | colon | hepatic flexure | 1.5cm |
| 6.8 |  | Sessile polyp | Not available | Colon | Mid descending colon | 2 |
| 6.8 |  | Sessile polyp | Not available | Colon | Rectum | 2 |
| 6.9 |  | Tubular adenoma, negative for high grade dysplasia | Not available | Colon | Sigmoid colon | diminutive |
| 6.9 |  | Tubular adenoma, negative for high grade dysplasia | Not available | Colon | mid transverse colon | 5 |
| 6.9 |  | Tubular adenoma, negative for high grade dysplasia | Not available | Colon | descending colon | 6 |

Lines with a number in the polyp ID column indicate samples where we had access to slides or blocks as well as a pathology report. For all of these samples two independent pathologists (LMW, VK) reviewed the samples and made their assessments. If they did not agree, they reviewed the material together again to reach a consensus.

**Supplementary Table 4:** **Tumour-derived RNA and DNA samples from Ox7 that were available for analysis**

| Sample name | Sample Type | Morphology | DNA analysis (MIP) | RNA analysis  (RNAseq) | Methylation analysis  (EPIC array) |
| --- | --- | --- | --- | --- | --- |
| RNF43_P1 | Frozen | Tubular adenoma | Yes | Yes | Yes |
| RNF43_P2 | Frozen | Sessile Serrated lesion | Yes | Yes | Yes |
| RNF43_P3 | Frozen | Unclassified polyp | No | Yes | No |
| RNF43_P4 | Frozen | Unclassified polyp | No | Yes | Yes |
| RNF43_P5 | Frozen | Sessile Serrated lesion | Yes | No | Yes |
| RNF43_P6 | FFPE | Sessile Serrated lesion | Yes | No | No |
| RNF43_P7 | FFPE | Tubular adenoma | Yes | No | No |
| RNF43_P8 | FFPE | Tubular adenoma | Yes | No | No |
| RNF43_P9 | FFPE | Traditional Serrated adenoma | Yes | No | No |

Nine polyps were obtained from Ox7 family member 5:16. Polyp type and number of analysis methods performed using each polyp is indicated. Microsatellite status was assessed by Genescan using multiplex PCRs amplifying regions containing mono or di nucleotide repeats. CIMP status was determined by calculating the mean beta for CpG islands annotated to the following genes and looking for divergent methylation patterns compared to a blood sample from the patient at CIMP panel loci: *CACNA1G, CDKN2A, CRABP1, IGF2, MLH1, NEUROG1, RUNX3* and *SOCS1.*

**Supplementary Table 5:** **Methylation beta values at CpGs annotated to *RNF43* in four polyps from Ox7:5:16 and a blood sample from the same patient**

| **probeID** | **Location** | **RNF43_P5** | **RNF43_P2** | **RNF43_P3** | **RNF43_P1** | **Blood DNA** |
| --- | --- | --- | --- | --- | --- | --- |
| cg05099390 | CGI;OpenSea | 0.028 | 0.024 | 0.025 | 0.022 | 0.025 |
| cg24835159 | CGI;OpenSea | 0.090 | 0.129 | 0.266 | 0.100 | 0.937 |
| cg08325901 | CGI;OpenSea | 0.079 | 0.163 | 0.235 | 0.069 | 0.760 |
| cg25450306 | CGI;OpenSea | 0.058 | 0.078 | 0.075 | 0.030 | 0.480 |
| cg22223182 | CGI;OpenSea | 0.124 | 0.152 | 0.298 | 0.116 | 0.669 |
| cg04544475 | CGI;OpenSea | 0.941 | 0.940 | 0.843 | 0.721 | 0.957 |
| cg05033557 | CGI;OpenSea | 0.283 | 0.320 | 0.268 | 0.097 | 0.885 |
| cg21917866 | CGI; Shore | 0.744 | 0.713 | 0.711 | 0.638 | 0.799 |
| cg07747280 | CGI;OpenSea | 0.118 | 0.157 | 0.304 | 0.109 | 0.814 |
| cg01912101 | CGI;OpenSea | 0.251 | 0.298 | 0.229 | 0.072 | 0.907 |
| cg11610460 | CGI; Shelf | 0.473 | 0.521 | 0.397 | 0.228 | 0.931 |
| cg02607754 | CGI;OpenSea | 0.025 | 0.022 | 0.020 | 0.011 | 0.040 |
| cg04654677 | CGI;OpenSea | 0.016 | 0.022 | 0.018 | 0.010 | 0.071 |
| cg04688486 | CGI;OpenSea | 0.354 | 0.405 | 0.291 | 0.164 | 0.949 |
| cg25452841 | CGI;OpenSea | 0.287 | 0.281 | 0.380 | 0.152 | 0.833 |
| cg18366480 | CGI;OpenSea | 0.324 | 0.451 | 0.337 | 0.232 | 0.837 |
| cg13583582 | CGI;OpenSea | 0.416 | 0.434 | 0.336 | 0.158 | 0.942 |
| cg02489371 | CGI;OpenSea | 0.132 | 0.208 | 0.276 | 0.075 | 0.781 |
| cg09120724 | CGI;OpenSea | 0.104 | 0.187 | 0.159 | 0.099 | 0.585 |
| cg04780984 | CGI;OpenSea | 0.131 | 0.184 | 0.289 | 0.106 | 0.969 |
| cg12558121 | CGI;OpenSea | 0.117 | 0.221 | 0.272 | 0.079 | 0.921 |
| cg15672877 | CGI;OpenSea | 0.119 | 0.158 | 0.303 | 0.114 | 0.890 |
| cg14398214 | CGI;OpenSea | 0.072 | 0.122 | 0.207 | 0.067 | 0.918 |
| cg21644778 | CGI;OpenSea | 0.382 | 0.469 | 0.360 | 0.114 | 0.776 |
| cg17885063 | CGI;OpenSea | 0.025 | 0.031 | 0.044 | 0.012 | 0.449 |
| cg04780629 | CGI;OpenSea | 0.018 | 0.018 | 0.016 | 0.013 | 0.010 |
| cg16473184 | CGI;OpenSea | 0.047 | 0.068 | 0.164 | 0.046 | 0.706 |

**Supplementary Table 6:** **Targets of the single molecule molecular inversion probe (smMIP) panel**

| *ACVR2A* | *BCL9L* | *FBXW7* | *MSH6* | *PTEN* | *SOX9* |
| --- | --- | --- | --- | --- | --- |
| *APC ** | *BMPR2* | *GNAS ** | *NRAS* | *RNF43* | *TCF7L2* |
| *ARID1A* | *BRAF ** | *KRAS* | *PIK3CA ** | *RPL22* | *TGIF1* |
| *ATM* | *CTNNB1 ** | *MLH1* | *POLD1* | *SMAD2* | *TP53* |
| *B2M* | *ELF3* | *MSH2* | *POLE* | *SMAD4* | *ZFP36L2* |

The smMIP panel was designed to target all exons and 10bp of flanking introns for genes not marked with *. For the genes marked by a * the following specific regions were targeted (positions are relative to hg19 of the human genome): *APC* codons 1-1600; *POLD1*: chr19:50905938-50910304; *POLE*: chr12:133249809-133253238; *GNAS* codon 200; *BRAF*: exons 11 and 15 (chr7: 140453074-140453193; chr7: 140481375_140481493; *CTNNB1* hotspots: chr3: 41266444-41266698; chr3: 41267150-41267352; chr3: 41274831-41274935; chr3: 41275019-41275358; chr3: 41277214-41277334; chr3: 41266016-41266244; *PIK3CA* hotspots: chr3: 178916613178916965; chr3: 178935997-178936122; chr3: 178951881-178952152). In total the panel targeted 63.1kb.

**Supplementary Table 7****: Primers used to assess MSI**

| Primer | Primer sequence | Multiplex PCR | Concentration (µM) |
| --- | --- | --- | --- |
| Bat25F | TCGCCTCCAAGAATGTAAGT | 1 | 8 |
| Bat25R | TCTGGATTTTAACTATGGCTC | 1 | 8 |
| Bat26F | TGACTACTTTTGACTTCAGCC | 1 | 4 |
| Bat26R | TTCTTCAGTATATGTCAATGAAAACA | 1 | 4 |
| D2S123F | AAACAGGATGCCTGCCTTTA | 1 | 8 |
| D2S123R | GGACTTTCCACCTATGGGAC | 1 | 8 |
| D5S346F | ACTCACTCTAGTGATAAATCGGG | 2 | 8 |
| D5S346R | AGCAGATAAGACAGTATTACTAGTT | 2 | 8 |
| D17S250F | GGAAGAATCAAATAGACAAT | 2 | 12 |
| D17S250R | GCTGGCCATATATATATTTAAACC | 2 | 12 |
| Bat40F | CCTACACCACAACCCTGCTT | 2 | 4 |
| Bat40R | TGAGGTGGGAGGATAAATGG | 2 | 4 |

Multiplex PCR 1: 10ul reactions were set up using 2x QIAGEN Multiplex PCR Master Mix and the concentrations indicated of each primer. 40ng of DNA was added to each reaction. PCR conditions used were: 15 min 95°C, followed by 35 cycles of 45 secs at 94°C, 1:30 min at 60°C, 45 secs at 72°C followed by a final extension step of 10 minutes at 72°C

Multiplex PCR 2: 10ul reactions were set up using 2x QIAGEN Multiplex PCR Master Mix and the concentrations indicated of each primer. 40ng of DNA was added to each reaction. PCR conditions used were: 15 min 95°C, followed by 35 cycles of 45 secs at 94°C, 1:30 min at 58°C, 45 secs at 72°C followed by a final extension step of 10 minutes at 72°C

**Supplementary Table 8:** **Mean beta values across all CpG islands annotated to genes commonly used to assign CpG Island Methylator Phenotype status**

| **Gene** | **RNF43_P5** | **RNF43_P2** | **RNF43_P3** | **RNF43_P1** | **Ox5:16** |
| --- | --- | --- | --- | --- | --- |
|  | Frozen SSL | Frozen SSL | Unclassified polyp | Frozen TA | Blood |
| *CACNA1G* | 0.852 | 0.863 | 0.795 | 0.713 | 0.937 |
| *CDKN2A* | 0.100 | 0.059 | 0.022 | 0.015 | 0.017 |
| *CRABP1* | 0.161 | 0.170 | 0.063 | 0.033 | 0.061 |
| *IGF2* | 0.355 | 0.435 | 0.184 | 0.214 | 0.113 |
| *MLH1* | 0.049 | 0.033 | 0.021 | 0.017 | 0.016 |
| *NEUROG1* | 0.406 | 0.486 | 0.148 | 0.192 | 0.112 |
| *RUNX3* | 0.473 | 0.551 | 0.379 | 0.332 | 0.602 |
| *SOCS1* | 0.133 | 0.148 | 0.093 | 0.095 | 0.113 |

All polyp samples showed similar patterns of methylation at these genes to those in DNA from the blood sample of that patient. The majority of the per gene beta values were <0.5, hence all four polyps were classified as CIMP-negative.

**Supplementary Table 9:** **Forty-three Wnt pathway genes included in the differential expression analysis presented in Supplementary Figure 3**

| *WNT3A* | *WNT6* | *WNT10B* | *FZD9* | *PLCB4* | *NKD2* | *MMP7* | *CHP2* | *LEF1* |
| --- | --- | --- | --- | --- | --- | --- | --- | --- |
| *SFRP5* | *CAMK2A* | *DKK1* | *WNT7B* | *AXIN2* | *WNT9A* | *APCDD1* | *CXXC4* | *DKK3* |
| *DKK2* | *CAMK2B* | *FZD2* | *FZD10* | *MYC* | *PRKCG* | *NKD1* | *VANGL2* | *PRICKLE2* |
| *WNT8B* | *SFRP4* | *DKK4* | *SFRP2* | *NOTUM* | *FOSL1* | *PLCB1* | *FZD7* |  |
| *WNT7A* | *SOX17* | *WNT2* | *SFRP1* | *WNT11* | *NFATC1* | *FZD3* | *WNT5A* |  |

**Supplementary Table 10:** **Phenotypes of individuals recruited to the CORGI study who underwent whole genome sequencing**

| Phenotype | N |
| --- | --- |
| CRC | 360 |
| CRC plus adenomas | 43 |
| CRC plus hyperplastic polyps | 13 |
| CRC plus mixed adenomas/polyps | 25 |
| Multiple adenomas | 18 |
| Multiple hyperplastic polyps | 33 |

**Supplementary Table 11****: Phenotypes of individuals recruited to the CORGI study or provided by collaborators who underwent whole exome sequencing**

| Phenotype | N |
| --- | --- |
| CRC | 169 |
| Multiple hyperplastic polyps | 51 |

**Supplementary Table 12: Coded cancer Types reported in UK Biobank and 100kGP participants that did not meet the selection criteria for controls.**

| Cancer Types diagnosed | N (UKB) | N (100kGP) | Germline LoF mutation in RNF43 |
| --- | --- | --- | --- |
| C34 (lung) | 1 | 1 | p.Pro659SerfsTer87 |
| C44 (skin) | 4 |  | p.Arg330Ter (N=2), p.Gly540ArgfsTer56, p. Glu590Ter |
| C44 (skin) and C54 endometrial | 1 |  | p.Pro659SerfsTer87 |
| C48 (peritoneum), C56 (ovary), C57.0 (Fallopian tube) | 1 |  | p.Val161SerfsTer7 |
| C49(connective and soft tissue) | 1 |  | p.Gly540ArgfsTer56 |
| C50 (breast) | 1 | 1 | p.Gln781Ter |
| C54 (endometrial) |  | 1 | p.Cys718LeufsTer28 |
| C61 (prostate) | 4 |  | p.Gln518SerfsTer9, p.Pro659SerfsTer87  p.Cys471ValfsTer31, |
| C65 (renal pelvis), C66 (ureter) and C67(bladder) | 1 |  | p.Arg330Ter |
| C66(ureter) | 1 |  | p.Arg371Ter |
| C67 (bladder) | 2 |  | p.Gln679Ter, p.Thr158ProfsTer6 |
| D06 (cervical cancer in situ) | 1 |  | p.Pro441ArgfsTer24 |

31 and 7 RNF43 LoF mutation carriers in UKB and 100kGP respectively had no ICD10 codes indicating cancer or pre-cancerous lesions, but were excluded from our control selection because of self-reported cancer (N=1 Hodkin lymphoma), family history of cancers in a first degree relative, missing data regarding cancer history of first degree relatives or in the case of 100KGP because of family history of a disease associated with an increased risk of cancer.   The remaining 18 in UKB and 3 in 100kGP had one or more ICD10-coded cancers or precursors. These phenotypes and genotypes of these participants are displayed in the table above.

**Supplementary Table 13.** **Distribution of 659fs genotypes deviates from that expected under no selection**

If somatic 659fs alleles were passengers, their genotypes should be present in proportion to a simple binomial distribution assuming independent mutations and no selection of the resultant genotypes, whether in the heterozygote state, as homozygotes or in combination with other (but pathogenic) *RNF43* alleles. We tested this prediction.

Analyses were based on MSI+ sporadic CRCs that had undergone WGS in the 100kGP (N=620). The observed numbers of CRCs with each of the three possible 659fs genotypes was counted. Cancers with uncertain zygosity were partitioned equally between the two possible genotypes, *e.g.* when 659fs status could not be assigned as homozygote or simple heterozygote (with the wildtype allele), a count of ½ was added to both the homozygous mutant and heterozygote totals. Similarly, the 659fs wildtype* homozygote total includes 306 MSI+ CRCs without *RNF43* mutations. Based on these data, 659fs allele frequency was calculated to be *p*_659fs_=0.270. Expected numbers of cancers with each genotype were calculated from the binomial:

N 659fs homozygotes = p^2^ x 620 ~ 45

N 659fs heterozygotes = 2p(1-p) x 620 ~ 244

N wildtype heterozygotes = (1-p)2 x 620 ~ 331

| 659fs genotype* | Observed no. tumours | Expected no. tumours | (Obs – Exp)^2^  Exp |  |
| --- | --- | --- | --- | --- |
| Homozygotes | 83 | 45 | 23.1 |  |
| Heterozygotes | 168 | 244 | 27.4 |  |
| Wildtype homozygotes | 359 | 331 | 3.8 |  |
| Total (N) | 620 | 620 | 54.3 | *P<10^-5^* |

*Includes a small number of cancers with other truncating mutations after codon 600

A χ^2^_1_ test comparing observed and expected numbers gives a statistic of 58.1, corresponding to *P*<10^-5^. There is thus evidence that 659fs homozygotes are present more often than expected by chance, plausibly because the genotype is selectively advantageous.

**Supplementary Table 14.** **Co-occurrence of 659fs/+ and 659fs/659fs genotypes with pathogenic *APC* or *CTNNB1* alleles differs from that expected under no selection of the 659fs mutation**

If 659fs (and other LoF mutations after *RNF43* codon 600) are passengers, the proportions of 659fs/+ and 659fs/659fs *RNF43* genotypes should be the same in cancers with or without pathogenic *APC* or *CTNNB1* genotypes. We tested this prediction (having excluded 659fs mutations of uncertain zygosity from the analysis). In fact, 659fs homozygosity was relatively more frequent in cancers without pathogenic *APC* or *CTNNB1* genotypes (*P*=0.022, Fisher’s exact).

|  | Pathogenic APC or CTNNB1 | No pathogenic APC or CTNNB1 | Total |
| --- | --- | --- | --- |
| 659fs/+ | 12 | 69 | 150 |
| 659fs/659fs | 2 | 62 | 79 |
| Total | 17 | 212 | 229 |

These data were consistent with selection for homozygous 659fs mutations in cancers that lacked Wnt activation by *APC* or *CTNNB1* mutations.

**Supplementary Table 15.** **Non-659fs mutations tend to occur with 659fs and other (non-659fs) *RNF43* mutations randomly when creating pathogenic genotypes.**

Here, we tested whether a 659fs allele fails to create a pathogenic genotype in combination with another, non-659fs LoF *RNF43* mutation, and hence is observed less often than expected compared with non-659fs mutations. The expected proportion of 659fs alleles was 0.688 and that of other loss-of-function *RNF43* alleles (“Other *RNF43*”) was (1-0.688)=0.312. The observed proportions of 659fs and “Other *RNF43*” alleles in combination with a single “Other *RNF43*” allele was very close to the numbers expected if 659fs is pathogenic (χ^2^_1_=1.00*, P=*0.32).

|  | Observed | Expected given relative allele frequency |
| --- | --- | --- |
| Other *RNF43* + 659fs | 77 | 73.5 |
| Other *RNF43* + Other *RNF43* | 28 | 31.5 |
| Total | 105 | 105 |

**Supplementary Table 16.** **Search for unsuspected Wnt pathway or other driver genes in *RNF43* 659fs*-*mutant CRCs.**

A hypothesis-free search was performed using the IntOGen pipeline [14] for driver mutations in the set of 162 sporadic CRCs (all bar one MSI+) from 100kGP, in which 659fs or other nearby mutations were the only *RNF43*-inactivating changes found. The table shows all drivers with combined Q<0.001. The third column shows which of the seven algorithms incorporated into the IntOGen pipeline individually found the gene to be a driver (MP=MutPanning, HM=HotMaps); this does not exclude supporting evidence from other algorithms; “Combination” here indicates that no single algorithm identified the gene as a driver, but that the combined evidence from all algorithms did so. The penultimate column is based on drivers reported by Cornish *et al* [15]; whilst *SFRP4* was not specifically identified as a driver in that study, it was noted as a possible target gene in a region of recurrent, significant copy number gain. Note that the mutations within *SFRP4* are predicted as protein-activating, but this classification is tentative and may not reflect the complexities of SFRP4 biology.

| **Driver gene** | **Q_combination_** | **Methods** | **Known?** | **Suggested function** |
| --- | --- | --- | --- | --- |
| *BRAF* | 9x10^-62^ | Multiple | Yes | Activating |
| *TP53* | 1x10^-35^ | Multiple | Yes | LoF |
| *FBXW7* | 4x10^-31^ | Multiple | Yes | LoF |
| *PIK3CA* | 4x10^-19^ | Multiple | Yes | LoF |
| *SOX9* | 3x10^-16^ | Multiple | Yes | Activating |
| *B2M* | 1x10^-15^ | Multiple | Yes | LoF |
| *ZNRF3* | 5x10^-15^ | MP | Yes | Ambiguous |
| *BMPR2* | 2x10^-10^ | Multiple | Yes | LoF |
| *PTEN* | 2x10^-9^ | Multiple | Yes | LoF |
| *CASP8* | 3x10^-8^ | Combination | Yes | LoF |
| *ARID2* | 9x10^-7^ | HM | Yes | Activating |
| *FAT4* | 2x10^-6^ | HM | Yes | LoF |
| *GRM3* | 3x10^-6^ | Combination | Yes | LoF |
| *GNAS* | 3x10^-6^ | MP | Yes | LoF |
| *KRAS* | 6x10^-5^ | HM | Yes | Activating |
| *SMAD4* | 1x10^-4^ | MP | Yes | Ambiguous |
| *GRINA* | 1x10^-4^ | Combination | No | Activating |
| *UBR5* | 4x10^-4^ | MP | Yes | Ambiguous |
| *SFRP4* | 5x10^-4^ | MP | No | Ambiguous |
| *MYO5A* | 5x10^-4^ | HM | No | LoF |
| *PPP2R1A* | 9x10^-4^ | Combination | No | LoF |

**Supplementary Figure 1 (related to Figure 3).** **Representative images of lesion types from Ox7 at three magnifications.** A-C sessile serrated lesion (SSL) with elongated crypts and serrated epithelium; D-F SSL with serrated dysplasia, low grade, cells with prominent nuclei showing pseudostratification and hypereosinophilic cytoplasm; G-I low grade SSL with intestinal dysplasia resembling that of conventional adenomas with tubular architecture; J-L traditional serrated adenoma (TSA), with characteristic ectopic crypts and generalised cytologic dysplasia; M-O tubular adenoma (TA) with hyperchromatic basal nuclei, showing surrounding disorganised crypts with low goblet cell density.

**
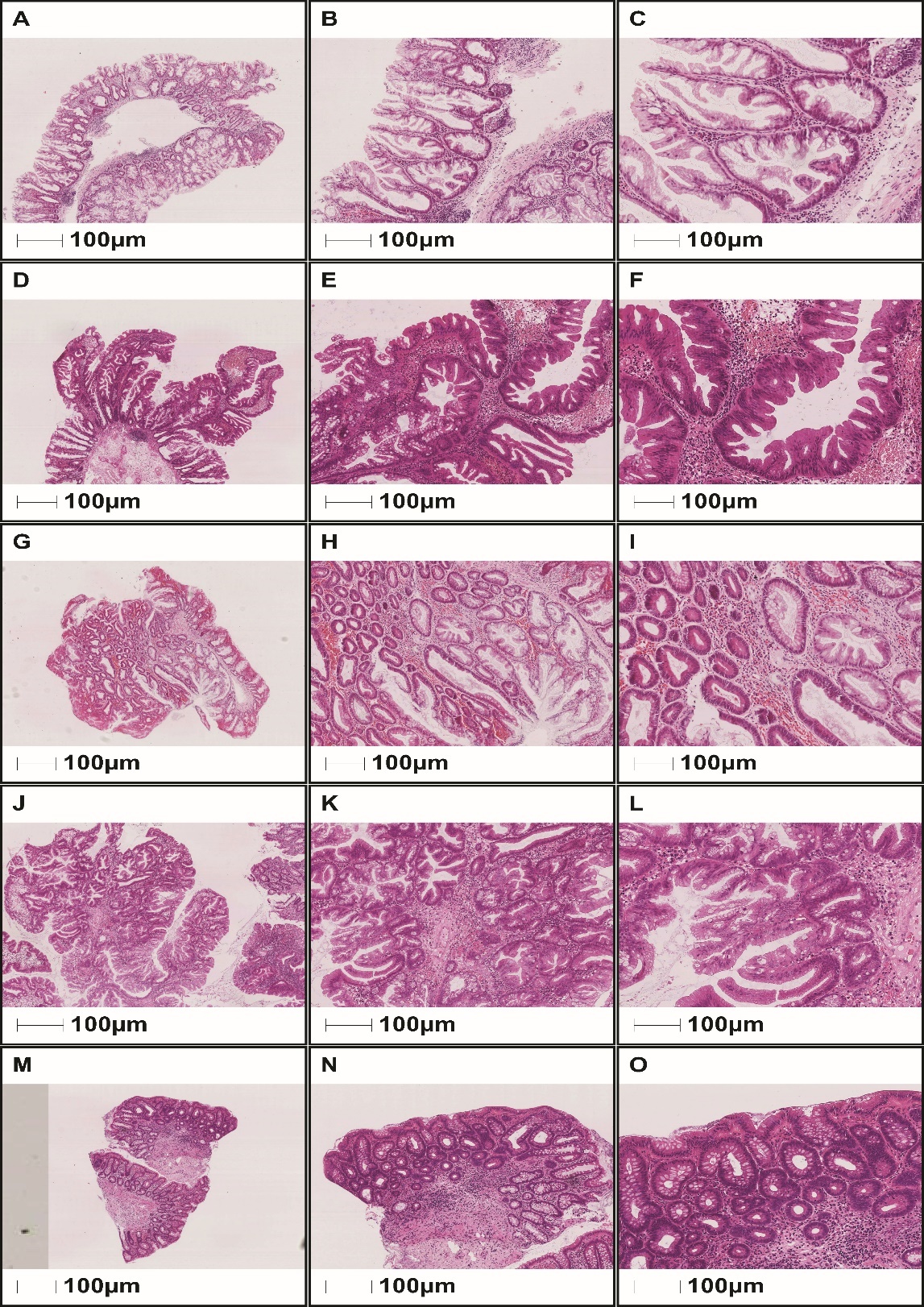
**

**Supplementary Figure 2.** **Somatic *RNF43, BRAF* and *KRAS* mutations detected in polyps from one individual of family Ox7.** Seven polyps were sequenced using single molecule molecular inversion probes targeting genes commonly mutated in CRC (**Supplementary Table 5**). All 7 polyps were microsatellite stable and CIMP (CpG island methylator phenotype)-negative.

**Supplementary Figure 3:** **Differential gene expression analysis of genes in the WNT pathway in four polyps from Ox7 and 53 sporadic polyps collected by the S:CORT study**


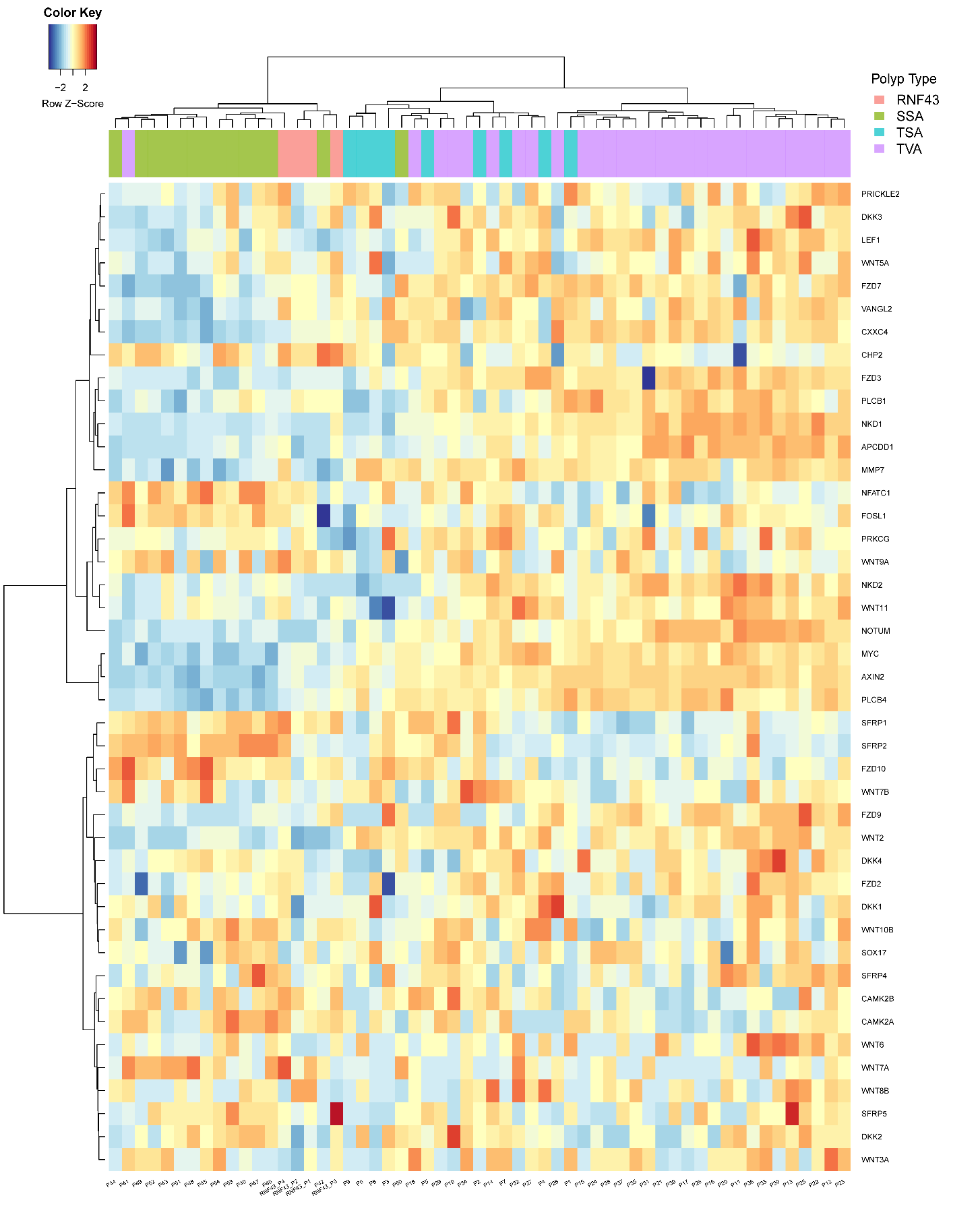


**Supplementary Figure 4:** **Box plots showing the expression of negative regulators of the Wnt pathway in polyps from Ox7 and 54 sporadic polyps collected by the S:CORT study.** Expression levels of negative regulators of the Wnt pathway have been shown to distinguish ligand dependent and ligand independent colorectal tumours.

**
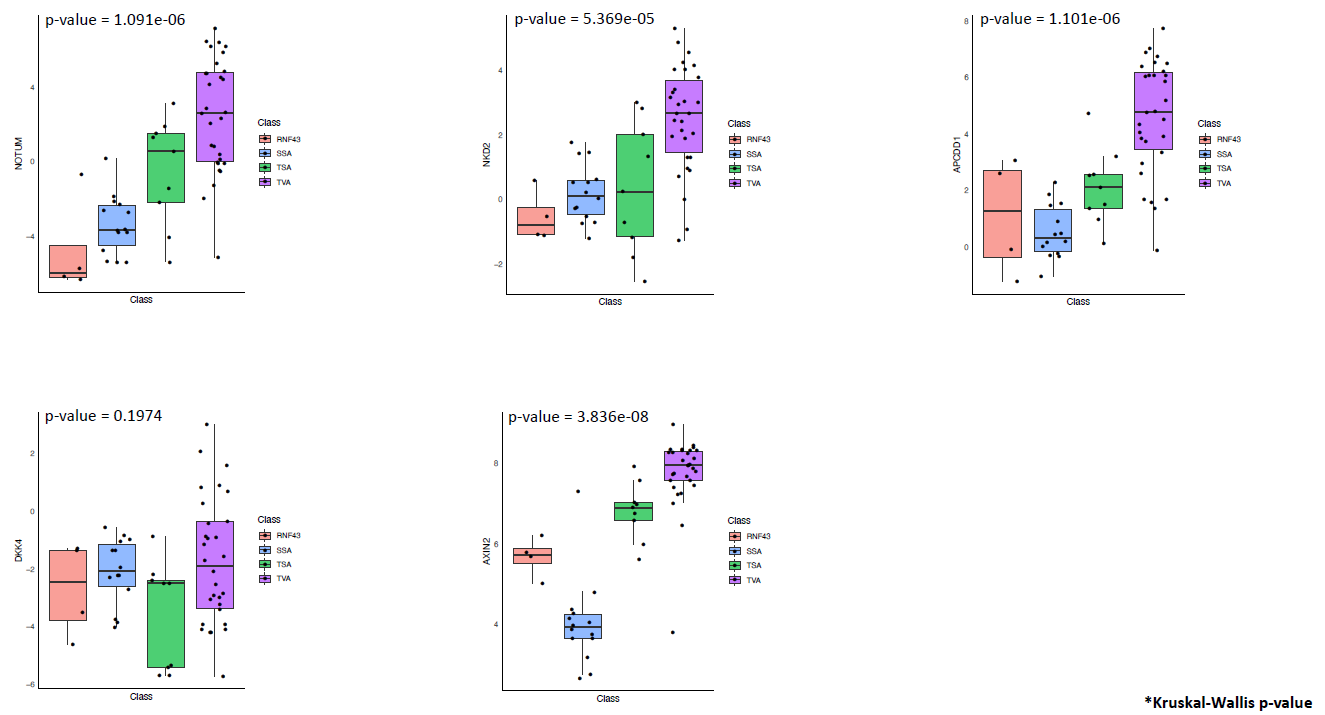
**

**Supplementary Figure 5.** **Breakdown of *RNF43-*mutant sporadic CRCs according to MSI and Wnt driver status.** The number of cancers with each of the 12 mutant genotype classes are shown.

**
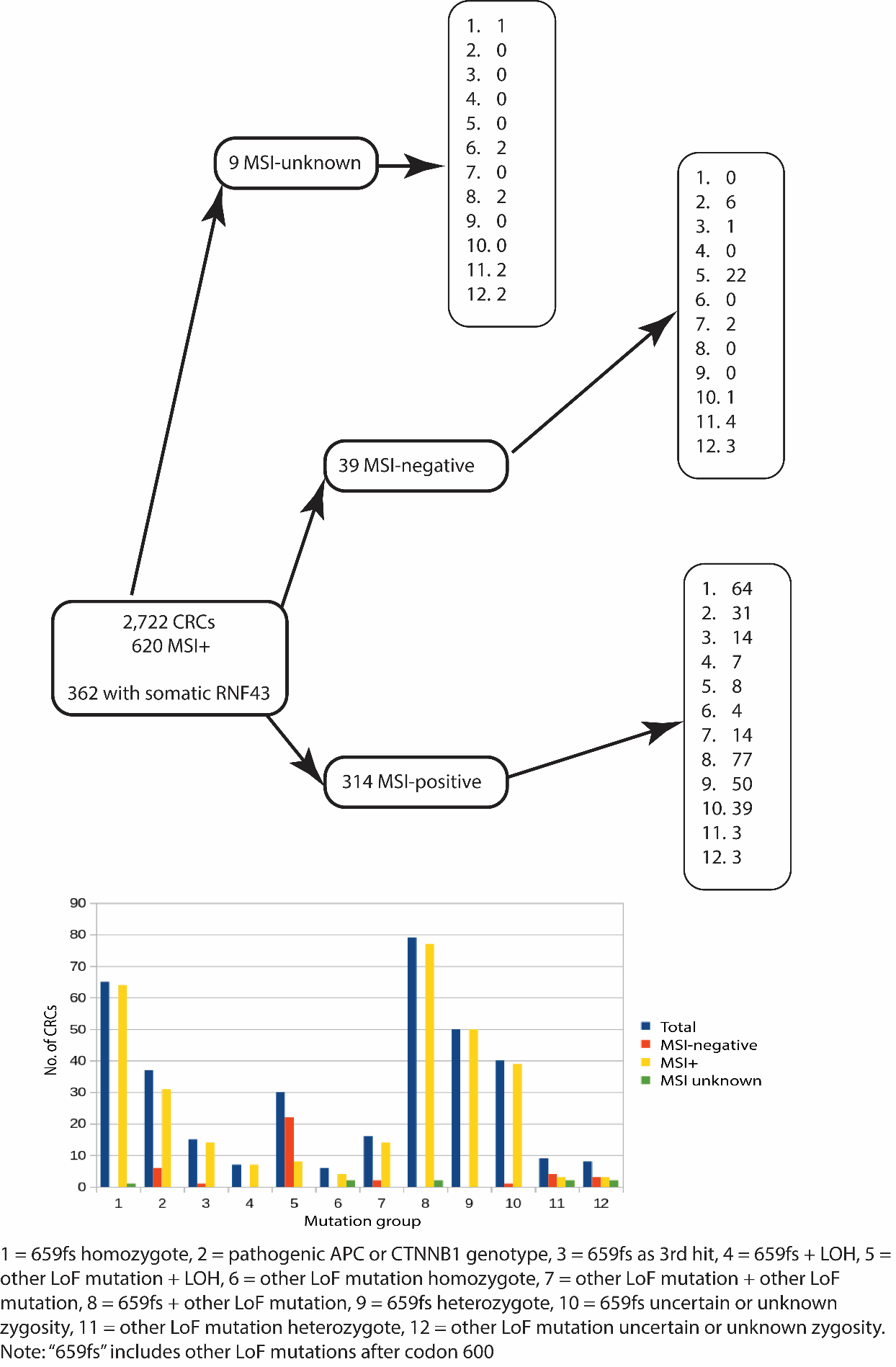
**

**Supplementary Figure 6.** **Nonsense-mediated decay assessment of mutant *RNF43* transcripts across 17 sporadic CRCs**. Almost all transcripts carrying premature truncating LoF variants were readily detectable in RNAseq data (details not shown). The tumours shown below had a single LoF allele (as heterozygote, homozygote or uncertain zygosity) in order to avoid confounding by putative second somatic mutations *in trans*. Read counts (mutant and reference) at the site of the mutation were compared in DNA and mRNA in a 2x2 format. Odds ratios (ORs) of mutant:reference alleles were calculated (OR>1 implies mutant more stable than expected in mRNA). The ORs are shown in ascending order. The three final tumours had no detectable wildtype allele counts in mRNA, implying monoallelic expression of the mutant. The apparent tendency for higher mutant allele expression was unexpected and unexplained but plausibly resulted in part from generally higher *RNF43* mRNA expression in tumour cells than contaminating ‘normal’ cells. In summary, loss of LoF-mutant transcripts through nonsense-mediated RNA decay does not appear to be the norm.

**Supplementary Figure 7.** ***AXIN2* mRNA expression in different sub-groups of CRCs with *RNF43* and other Wnt driver genotypes.**

|  | **Group 0** | **Group 1** | **Finding** |
| --- | --- | --- | --- |
| A | RNF43-wildtype (most APC) | RNF43-mutant CRCs in this study | Confirms much higher AXIN2 with APC |
| B | Pathogenic APC genotype (plus single LoF RNF43 allele) | Bi-allelic (homozygous) RNF43 659fs | Much higher AXIN2 with APC even with RNF43 passenger present |
| C | Non-659fs bi-allelic LoF RNF43 mutations | Bi-allelic (homozygous) RNF43 659fs | 659fs bi-allelic has no evidence of other ligand-independent Wnt driver |
| D | Heterozygous 659fs | Bi-allelic (homozygous) RNF43 659fs | Perhaps small increase if RNF43 inactive |
| E | Non-659fs bi-allelic LoF RNF43 mutations | 659fs + non-659fs LoF RNF43 mutation | 659fs similar to other mutations |
| F | Non-659fs bi-allelic LoF RNF43 mutations | Mono-allelic (heterozygous with wildtype) 659fs | Perhaps small increase if RNF43 inactive |


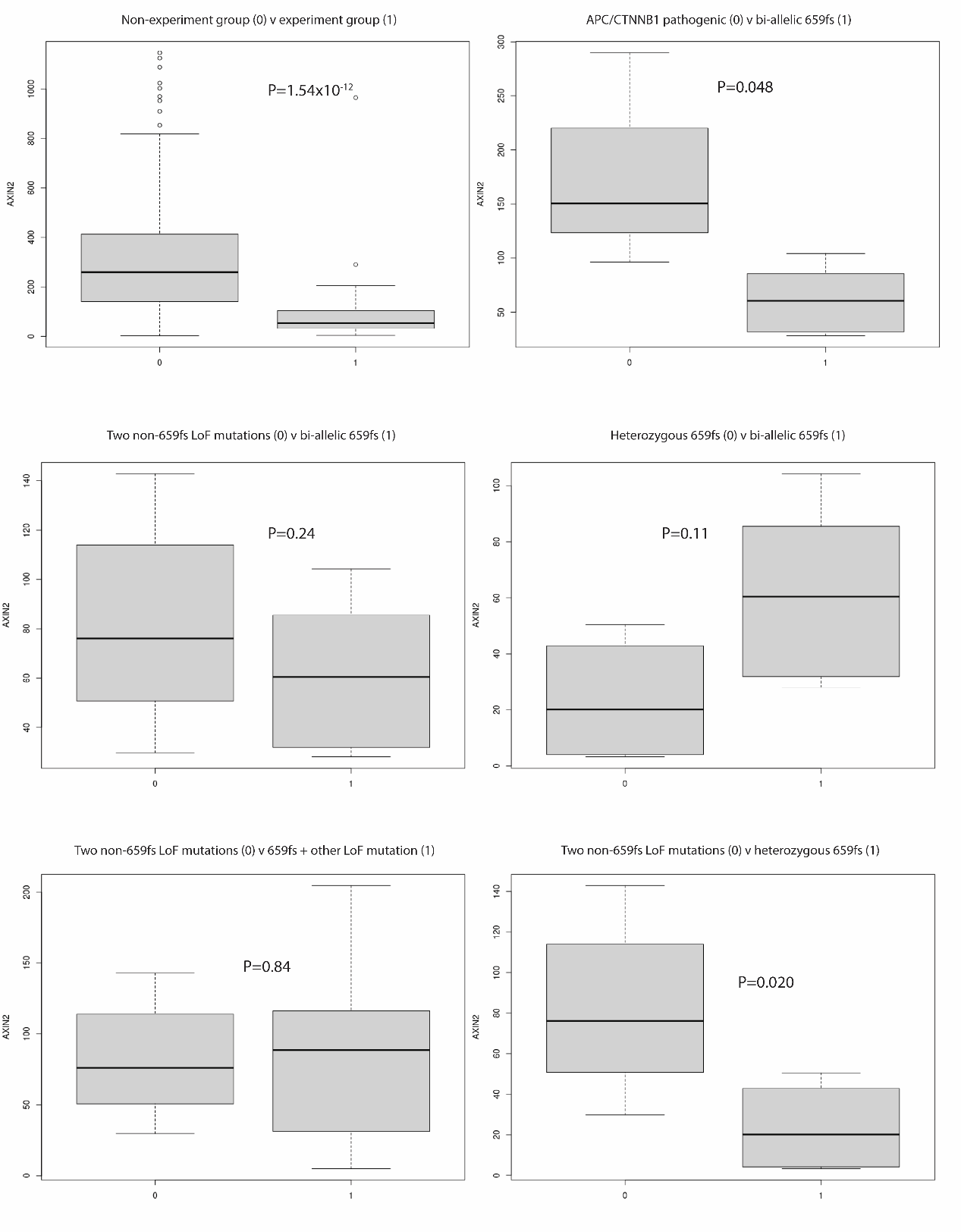


B

D

F

A

C

E

**Supplementary Figure 8.** **Pairwise association between Wnt driver mutations by gene in MSI+ CRCs.**

Positive association is shown in blue, with association ln(odds ratio) from a regression model taking into account age, sex and tumour location in each square. * P<0.05, ** P<0.01, *** P<0.001. The association between *RNF43* and *ZNRF3* mutations is outlined and the strongest co-occurrence detected.


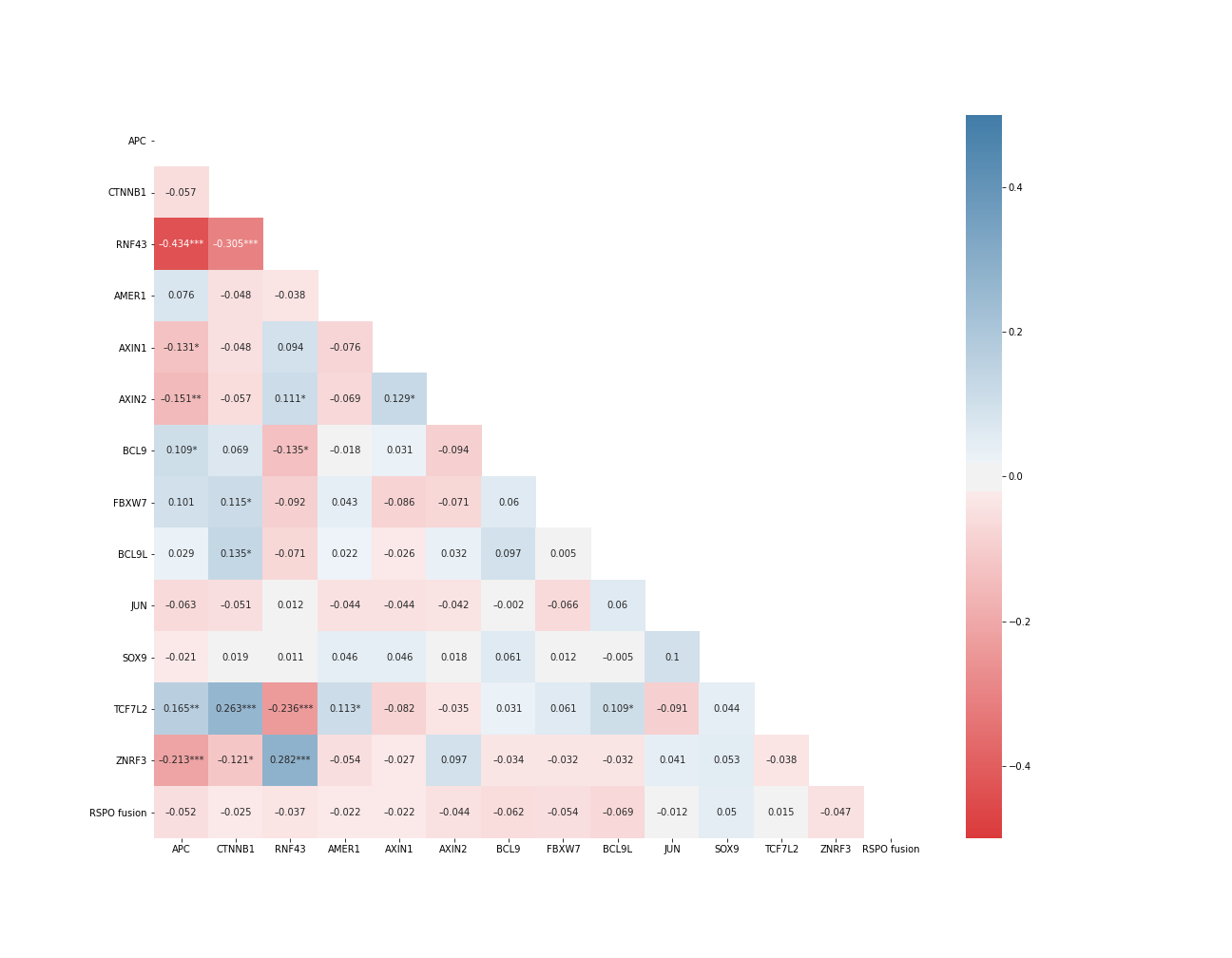


**Supplementary Methods**

*Whole genome sequencing (WGS) of patients with CRC or colorectal polyposis*

Whole genome sequencing of genomic DNA (gDNA) was performed using Illumina 75bp paired end sequencing to 30x depth or Complete Genomics technology (mean average coverage 58x) (**Supplementary Tables 10 & 11**). Genomes sequenced on these two different platforms were analysed separately. For the Illumina sequenced samples, FASTQ files were mapped to build 37 (hg19) of the human genome reference using an in-house pipeline making use of SAMtools [16], BEDtools [17], fastqutils [18], BWA aln [19], STAMPY [20] and the picard suite of tools (http://broadinstitute.github.io/picard/). Deduplicated BAM files were then submitted to Genome Analysis Tool Kit (GATK) pre-processing for variant discovery steps: IndelRealigner, BaseRecalibrator, PrintReads. HaplotypeCaller [21] was used in GVCF mode to call variants in per sample mode using the analysis ready BAMs. CombineGVCFs was used to generate per chromosome multi-sample GVCFs. Variants were then joint called in discovery mode using a minimum phred-scaled Q score confidence threshold of 10 for emission and of 30 for high-confidence calling. Variant Quality Score Recalibration (VQSR) was used to filter the raw variant calls with all variants in the 99.9% and above tranches being retained. Variant tools (VT) [22] was used in smart mode to decompose variants to biallelic sites and variant annotation was performed using the Ensembl Variant Effect Predictor (VEP) framework [23]. Frequency information for each variation was obtained from the following sources: 1) 144 genomes from patients with non-cancer phenotypes sequenced as part of the WGS500 project version 14 [24], 2) UK10K [25], 3) ExAC [26] and 4) 1000 genomes project [27]. An additional 13 germline genomes sequenced using Complete Genomics Technology, generated as part of paired tumour-normal sequencing of colorectal cancers, were also analysed [28]. 1µg of DNA from 6.5 was analysed by whole genome sequencing at Novogene (Cambridge). The average read depth was 26.9. Reads were mapped using BWA mem (v0.7.17) [29] before following BAM file processing and germline variant calling as described above.

*Exome sequencing of patients with CRC or colorectal polyposis*

Exome sequencing was performed either as previously described (DNAs from patients with CRC) [30] or using Sanger Institute custom exome sequencing baits to an average depth of 65x (DNAs from patients with multiple serrated polyps). One of the samples analysed by exome sequencing was a relative of another analysed by WGS. All other samples were unrelated. Variants with a call rate of less than 75% were excluded. We prioritised variants that were rare in controls, keeping only those with ≤2 observations in WGS500 cancer free controls ≤100 counts in ExAC. Variant Effect Predictor (VEP) plugin LoF transcript effect estimator (LOFTEE) was used to identify high and low confidence LoF variants (that is, predicted null or protein-truncating) and only these variants were retained. Where genome or exome calls were available for multiple affected individuals from the same family, variants identified in all related individuals were extracted using information on pattern of inheritance to prioritise heterozygous or homozygous calls. VEP was also used to annotate variants identified across all the sample sets described above with variant impact classifications including SIFT and polyphen scores and information regarding pathogenicity from ClinVar [31].

*DNA and RNA extraction from polyps*

DNA was extracted from available FFPE and fresh frozen polyps. FFPE tumour DNA was extracted from 8µm sections using the Roche HighPure FFPE extraction kit following overnight incubation with proteinase K. DNA and RNA was extracted from frozen polyps (stored in LN until time of extraction) using AllPrep DNA/RNA Mini Kit (Qiagen). Samples were homogenised using a pestle and mortar and those under 30mg were passed through a QIAshredder (Qiagen), while those greater than 30mg were passed repeatedly through a blunt needle and syringe until no clumps of tissue were visible. Manufacturer’s instructions were then followed with samples greater than 30mg split into two columns. DNA was quantified using Qubit. RNA was DNAse treated, quantified using Qubit, and stored at -80˚C. DNA from blood was available from this patient and was used as a normal control in DNA sequencing and methylation analysis.

*RNA sequencing from fresh frozen polyps*

RNA sequencing was performed using TruSeq Stranded Total RNA Library Prep kit (Illumina) with H/M/R Gold probes. Pooled libraries were sequenced using 75bp paired end reads on HiSeq4000 aiming for 50 million reads. Fastq reads were quality checked by trimming for low-quality ends and clipping adapter sequences if present, using Trimmomatic (v.0.36) [32]. QC processed reads were then mapped to the human genome (GRCh37) and transcriptome (Ensembl release 87) using STAR aligner (v. 2.6.2b) two-pass mode [33]. Gene level count data thus obtained were subsequently normalised using the 'voom' method and differential gene-expression comparisons using the empirical Bayes method implemented in limma package (v.3.44.1) in R (v.3.4.0) [34]. Wnt pathway specific analysis was performed by accessing the KEGG_WNT_SIGNALING_PATHWAY from MSigDb (**Supplementary Table 9**). We also investigated the expression of genes proposed for distinguishing Wnt ligand independent and Wnt ligand dependent tumours . A data set of 53 sporadic colorectal adenomas – including 9 traditional serrated adenomas (TSA), 14 sessile serrated lesions (SSL) and 30 tubulo-villous adenomas (TVA) – from the S:CORT consortium was sequenced using the same library prep kit and sequencing platform was used for the differential gene expression analysis. One patient had a presumed somatic LoF variant in *RNF43 (*p.Arg337Ter) called from the adenoma sample and this sample was excluded from analyses.

*Targeted DNA sequencing from FFPE and fresh frozen polyps*

FFPE and fresh frozen polyps with sufficient DNA were analysed using a custom single molecule molecular inversion probe panel (smMIP) targeting 30 genes commonly mutated in colorectal cancers, including *RNF43, BRAF, MSH2, KRAS, APC, BCL9L, TP53,* and *NRAS* (full details in **Supplementary Table 6**). Library preparation proceeded as described and was attempted for all samples where DNA extraction yielded > 50ng. Libraries were indexed and sequenced on the Nextseq (Illumina) using custom sequencing primers aiming for 1000x sequencing depth of tumours and 100x of paired normal (blood). Reads were processed with MIPGEN tools [35] and aligned to hg19 using BWA mem [29]. Variants were consensus called following variant calling using lofreq [36]. LOH at *RNF43* was assessed using common coding SNPs targeted by smMIPs.

*Methylation analysis of polyps and normal blood samples*

250ng of DNA from FFPE, fresh frozen polyps and blood was analysed on the Illumina Methylation array. DNA from FFPE samples underwent the recommended DNA repair step. Raw analysis files were processed using the CHAMP pipeline [37]. In order to assess the CpG island methylator phenotype (CIMP), the methylation beta values for CpGs within islands annotated to the following genes were obtained and the mean of these betas was calculated for each sample (*CACNA1G*, *CDKN2A*, *CRABP1*, *IGF2*, *MLH1*, *NEUROG1*, *RUNX3*, *SOCS1*) (**Supplementary Table 8**). We also checked for silencing of *RNF43* by methylation by comparing the methylation levels at CpGs (0 islands, 1 shore, 1 shelf and 25 sea) annotated to *RNF43* in the blood sample and in the polyps (**Supplementary Table 5**).

*Microsatellite instability analysis by PCR*

Primers used to amplify mononucleotide and dinucleotide repeats can be found in **Supplementary Table 7**. All polyp samples and matched blood samples were amplified using the Qiagen multiplex PCR kit multiplex PCR using the conditions provided. Products were visualised on a 1% agarose gel and diluted 1 in 20 before being submitted for genescan analysis. Results from polyps and matched normal were visualised using Gene Marker V3.0.1 (SoftGenetics) and polyps assessed for additional repeats not observed in the normal sample.

*Variant annotation and pathogenicity assignment*

Owing to uncertainty regarding the pathogenicity of some types of *RNF43* mutation, we only considered as pathogenic frameshift, nonsense and splice site (LoF) mutations classed as pathogenic by spliceAI (score ≥0.80). Whilst we reported ClinVar pathogenicity annotations for germline and somatic variants of interest, that annotation has inherent challenges. Specifically, we regarded the small proportion of pathogenic missense mutations in ClinVar as variants of uncertain significance (VUSs) [31]. For *APC,* we regarded truncating mutations between codons 70 and 1600 as pathogenic, and bi-allelic mutations (including copy-neutral or deletion LOH) as creating a pathogenic genotype. For *CTNNB1,* we regarded heterozygous point mutations or in-frame deletions of critical phosphorylation sites in exon 3 as pathogenic. Where more than one pathogenic mutation was present in the same gene, we assumed that those were present *in trans* in the absence of information to the contrary.

*Calling microsatellite instability, copy number changes and loss of heterozygosity from tumour WGS in 100kGP*

MSIngs was used to call microsatellite instability, supplemented in a small number of equivocal cases by mutation burden assessment and COSMIC signature analysis [38]. Somatic copy number and loss-of-heterozygosity (LOH) analyses were performed using the Battenberg program [39], supplemented by the Sequenza program [40] where Battenberg produced equivocal results. All copy number data for the *RNF43* and *APC* loci were inspected visually to confirm automated calls.

**Supplementary References**

1 Gala MK, Mizukami Y, Le LP, Moriichi K, Austin T, Yamamoto M*, et al.* Germline mutations in oncogene-induced senescence pathways are associated with multiple sessile serrated adenomas. Gastroenterology 2014;**146**:520-9.

2 Taupin D, Lam W, Rangiah D, McCallum L, Whittle B, Zhang Y*, et al.* A deleterious RNF43 germline mutation in a severely affected serrated polyposis kindred. Human genome variation 2015;**2**:15013.

3 Yan HHN, Lai JCW, Ho SL, Leung WK, Law WL, Lee JFY*, et al.* RNF43 germline and somatic mutation in serrated neoplasia pathway and its association with BRAF mutation. Gut 2017;**66**:1645-56.

4 Quintana I, Mejías-Luque R, Terradas M, Navarro M, Piñol V, Mur P*, et al.* Evidence suggests that germline RNF43 mutations are a rare cause of serrated polyposis. Gut 2018;**67**:2230-2.

5 Mikaeel RR, Young JP, Li Y, Poplawski NK, Smith E, Horsnell M*, et al.* RNF43 pathogenic Germline variant in a family with colorectal cancer. Clinical genetics 2022;**101**:122-6.

6 Chan JM, Clendenning M, Joseland S, Georgeson P, Mahmood K, Joo JE*, et al.* Inherited BRCA1 and RNF43 pathogenic variants in a familial colorectal cancer type X family. Familial cancer 2024;**23**:9-21.

7 Brinch HH, Byrjalsen A, Lohse Z, Rasmussen A, Karstensen JG, Kristiansen BS*, et al.* Germline pathogenic variants in RNF43 in patients with and without serrated polyposis syndrome. Familial cancer 2024;**24**:3.

8 Cho AR, Sul HJ, Kim YJ, Kim B, Zang DY. RNF43 R117fs mutant positively regulates Wnt/β-catenin signaling by failing to internalize FZD expressed on the cell surface. Scientific reports 2022;**12**:7013.

9 Li S, Lavrijsen M, Bakker A, Magierowski M, Magierowska K, Liu P*, et al.* Commonly observed RNF43 mutations retain functionality in attenuating Wnt/β-catenin signaling and unlikely confer Wnt-dependency onto colorectal cancers. Oncogene 2020;**39**:3458-72.

10 Tu J, Park S, Yu W, Zhang S, Wu L, Carmon K*, et al.* The most common RNF43 mutant G659Vfs*41 is fully functional in inhibiting Wnt signaling and unlikely to play a role in tumorigenesis. Scientific reports 2019;**9**:18557.

11 Yu J, Yusoff PAM, Woutersen DTJ, Goh P, Harmston N, Smits R*, et al.* The Functional Landscape of Patient-Derived RNF43 Mutations Predicts Sensitivity to Wnt Inhibition. Cancer research 2020;**80**:5619-32.

12 Fang L, Ford-Roshon D, Russo M, O'Brien C, Xiong X, Gurjao C*, et al.* RNF43 G659fs is an oncogenic colorectal cancer mutation and sensitizes tumor cells to PI3K/mTOR inhibition. Nature communications 2022;**13**:3181.

13 Murphy A, Solomons J, Risby P, Gabriel J, Bedenham T, Johnson M*, et al.* Germline variant testing in serrated polyposis syndrome. Journal of gastroenterology and hepatology 2022;**37**:861-9.

14 Martínez-Jiménez F, Muiños F, Sentís I, Deu-Pons J, Reyes-Salazar I, Arnedo-Pac C*, et al.* A compendium of mutational cancer driver genes. Nat Rev Cancer 2020;**20**:555-72.

15 Cornish AJ, Gruber AJ, Kinnersley B, Chubb D, Frangou A, Caravagna G*, et al.* The genomic landscape of 2,023 colorectal cancers. Nature 2024;**633**:127-36.

16 Danecek P, Bonfield JK, Liddle J, Marshall J, Ohan V, Pollard MO*, et al.* Twelve years of SAMtools and BCFtools. Gigascience 2021;**10**.

17 Quinlan AR, Hall IM. BEDTools: a flexible suite of utilities for comparing genomic features. Bioinformatics 2010;**26**:841-2.

18 Breese MR, Liu Y. NGSUtils: a software suite for analyzing and manipulating next-generation sequencing datasets. Bioinformatics 2013;**29**:494-6.

19 Li H, Durbin R. Fast and accurate long-read alignment with Burrows-Wheeler transform. Bioinformatics 2010;**26**:589-95.

20 Lunter G, Goodson M. Stampy: a statistical algorithm for sensitive and fast mapping of Illumina sequence reads. Genome Res 2011;**21**:936-9.

21 Poplin R, Ruano-Rubio V, DePristo MA, Fennell T, J., Carneiro MO, Auwera GA*, et al.* Scaling accurate genetic variant discovery to tens of thousands of samples. BioRXiv 2015;**Pre-print**.

22 Tan A, Abecasis GR, Kang HM. Unified representation of genetic variants. Bioinformatics 2015;**31**:2202-4.

23 McLaren W, Gil L, Hunt SE, Riat HS, Ritchie GR, Thormann A*, et al.* The Ensembl Variant Effect Predictor. Genome Biol 2016;**17**:122.

24 Taylor JC, Martin HC, Lise S, Broxholme J, Cazier JB, Rimmer A*, et al.* Factors influencing success of clinical genome sequencing across a broad spectrum of disorders. Nature genetics 2015;**47**:717-26.

25 Walter K, Min JL, Huang J, Crooks L, Memari Y, McCarthy S*, et al.* The UK10K project identifies rare variants in health and disease. Nature 2015;**526**:82-90.

26 Lek M, Karczewski KJ, Minikel EV, Samocha KE, Banks E, Fennell T*, et al.* Analysis of protein-coding genetic variation in 60,706 humans. Nature 2016;**536**:285-91.

27 Fairley S, Lowy-Gallego E, Perry E, Flicek P. The International Genome Sample Resource (IGSR) collection of open human genomic variation resources. Nucleic Acids Res 2020;**48**:D941-d7.

28 Chubb D, Broderick P, Dobbins SE, Frampton M, Kinnersley B, Penegar S*, et al.* Rare disruptive mutations and their contribution to the heritable risk of colorectal cancer. Nature communications 2016;**7**:11883.

29 Li H. Aligning sequence reads, clone sequences and assembly contigs with BWA-MEM. arXiv 2013;**Pre-print**.

30 Smith CG, Naven M, Harris R, Colley J, West H, Li N*, et al.* Exome resequencing identifies potential tumor-suppressor genes that predispose to colorectal cancer. Hum Mutat 2013;**34**:1026-34.

31 Landrum MJ, Lee JM, Benson M, Brown GR, Chao C, Chitipiralla S*, et al.* ClinVar: improving access to variant interpretations and supporting evidence. Nucleic Acids Res 2018;**46**:D1062-d7.

32 Bolger AM, Lohse M, Usadel B. Trimmomatic: a flexible trimmer for Illumina sequence data. Bioinformatics 2014;**30**:2114-20.

33 Dobin A, Davis CA, Schlesinger F, Drenkow J, Zaleski C, Jha S*, et al.* STAR: ultrafast universal RNA-seq aligner. Bioinformatics 2013;**29**:15-21.

34 Ritchie ME, Phipson B, Wu D, Hu Y, Law CW, Shi W*, et al.* limma powers differential expression analyses for RNA-sequencing and microarray studies. Nucleic Acids Res 2015;**43**:e47.

35 Boyle EA, O'Roak BJ, Martin BK, Kumar A, Shendure J. MIPgen: optimized modeling and design of molecular inversion probes for targeted resequencing. Bioinformatics 2014;**30**:2670-2.

36 Wilm A, Aw PP, Bertrand D, Yeo GH, Ong SH, Wong CH*, et al.* LoFreq: a sequence-quality aware, ultra-sensitive variant caller for uncovering cell-population heterogeneity from high-throughput sequencing datasets. Nucleic Acids Res 2012;**40**:11189-201.

37 Tian Y, Morris TJ, Webster AP, Yang Z, Beck S, Feber A*, et al.* ChAMP: updated methylation analysis pipeline for Illumina BeadChips. Bioinformatics 2017;**33**:3982-4.

38 Alexandrov LB, Jones PH, Wedge DC, Sale JE, Campbell PJ, Nik-Zainal S*, et al.* Clock-like mutational processes in human somatic cells. Nature genetics 2015;**47**:1402-7.

39 Nik-Zainal S, Alexandrov LB, Wedge DC, Van Loo P, Greenman CD, Raine K*, et al.* Mutational processes molding the genomes of 21 breast cancers. Cell 2012;**149**:979-93.

40 Favero F, Joshi T, Marquard AM, Birkbak NJ, Krzystanek M, Li Q*, et al.* Sequenza: allele-specific copy number and mutation profiles from tumor sequencing data. Ann Oncol 2015;**26**:64-70.
