# Supplementary material for: Comparison between germline and somatic loss-of-function RNF43 mutations reveals different genotype-phenotype associations and provides insights into the genetic mechanisms of colorectal tumourigenesis
Source: Gut. 2025 Dec 24;75(7):e337030. doi: 10.1136/gutjnl-2025-337030 (PMC13311953; doi:10.1136/gutjnl-2025-337030)
Supplement: online supplemental file 2 [file gutjnl-75-7-s002.pptx]

## Slide 1
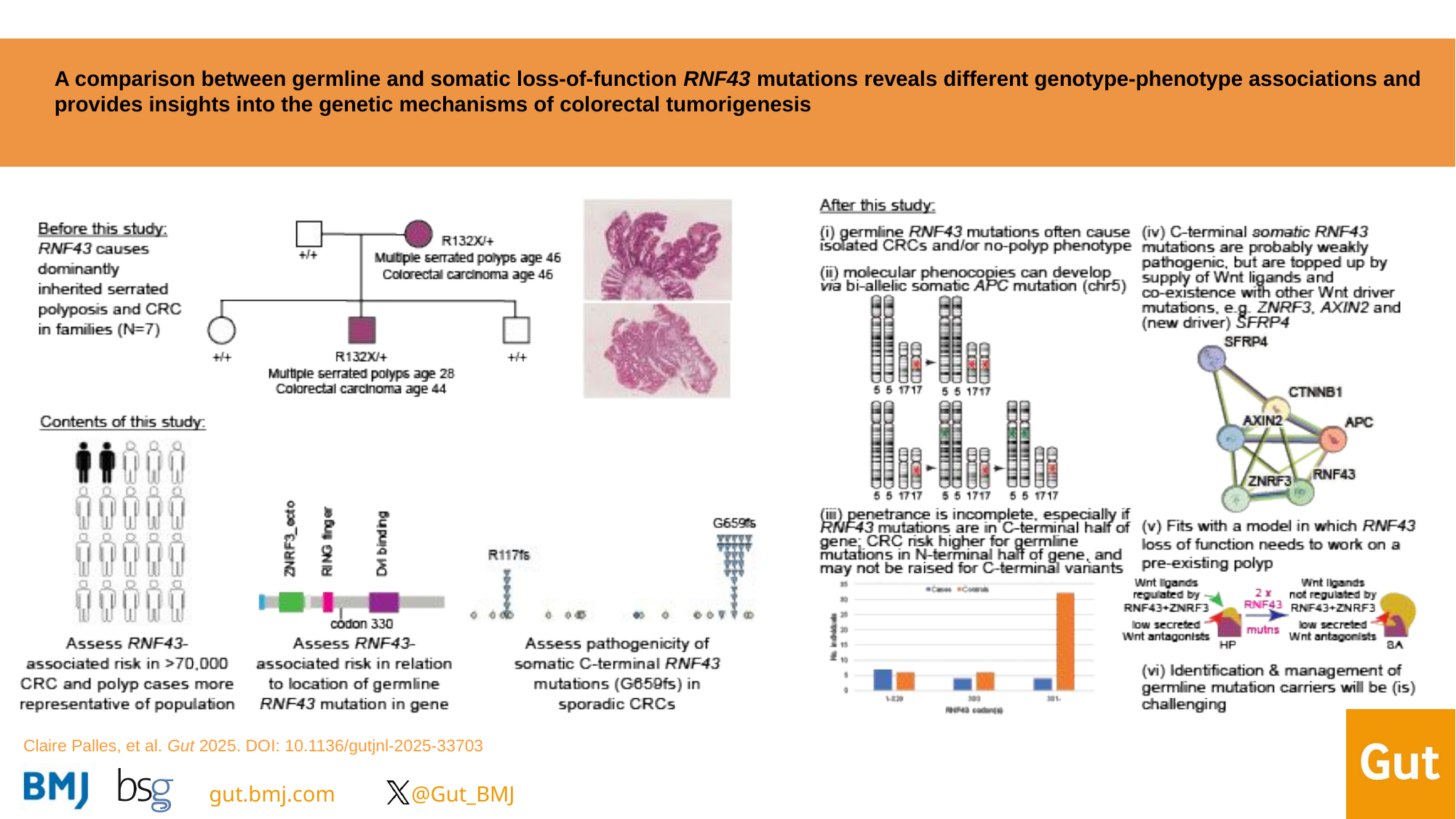

A comparison between germline and somatic loss-of-function RNF43 mutations reveals different genotype-phenotype associations and provides insights into the genetic mechanisms of colorectal tumorigenesis
Claire Palles, et al. Gut 2025. DOI: 10.1136/gutjnl-2025-33703
gut.bmj.com
@Gut_BMJ
